# Optically driven plasmons in graphene/hBN van der Waals heterostructures: simulating s-SNOM measurements

**DOI:** 10.1515/nanoph-2023-0841

**Published:** 2024-04-15

**Authors:** Neven Golenić, Stefano de Gironcoli, Vito Despoja

**Affiliations:** Department of Physics, University of Zagreb, Bijenička 32, 10000, Zagreb, Croatia; 19040 Scuola Internazionale Superiore di Studi Avanzati (SISSA), Via Bonomea 265, 34136 Trieste, Italy; Centre for Advanced Laser Techniques, Institute of Physics, Bijenička 46, 10000 Zagreb, Croatia; Donostia International Physics Center (DIPC), P. Manuel de Lardizabal, 4, 20018 San Sebastián, Spain; CNR-IOM DEMOCRITOS, Istituto Officina dei Materiali, Trieste, Italy

**Keywords:** s-SNOM, graphene, hBN, photons, optical excitations, Dirac plasmon polariton

## Abstract

Converting transverse photons into longitudinal two-dimensional plasmon-–polaritons (2D-PP) and vice versa presents a significant challenge within the fields of photonics and plasmonics. Therefore, understanding the mechanism which increases the photon – 2D-PP conversion efficiency could significantly contribute to those efforts. In this study, we theoretically examine how efficiently incident radiation, when scattered by a silver spherical nanoparticle (Ag-NP), can be transformed into 2D-PP within van der Waals (vdW) heterostructures composed of hexagonal boron nitride and graphene (hBN/Gr composites). We show that the Dirac plasmon (DP) excitation efficiency depends on the Ag-NP radius as *R* ^3^, and decreases exponentially with Ag-NP height *h*, so that for a certain Ag-NP geometry up to 25 % of the incident electrical field is channeled into the DP. We demonstrate that the linear plasmons (LPs) excitation efficiency can be manipulated by changing the graphene–graphene distance Δ (or hBN thickness) or by changing the number of graphene layers *N*. By increasing Δ and/or *N* the LPs move towards smaller wave vectors *Q* and become accessible by the Ag-NP dipole field, so that for *N* ≥ 5 the excitation of more than one LP is possible. These results are supported by recent scattering-type scanning near-field optical microscopy (s-SNOM) measurements. Furthermore, we show that Ag-NPs with specific parameters preferentially hybridizes with DPs of a particular wavelength *λ*_*D*_, facilitating selective excitation of DPs. The obtained tuning possibilities could have a significant impact on applied plasmonics, photonics or optoelectronics.

## 1 Introduction

Longitudinal collective electromagnetic modes in atomically thin 2D crystals known as 2D plasmon–polaritons (2D-PP) were extensively studied in the last decade. Namely, 2D-PPs carry a strong evanescent electric field that propagates only close to the 2D crystal, hence their trajectory along the 2D crystal can be efficiently manipulated akin to a *nanoscale waveguide* [1]. Even more, the intensity, frequency and range of 2D-PPs strongly depend on the effective number of charge carriers in the valence band, which in 2D crystals can be changed electrostatically but also using different types of 2D crystals. These properties have facilitated the use of conductive 2D crystals in numerous applications across optoelectronics, photonics [1], [2], [3], plasmonics [4], [5], [6], as a photodetectors, sensors and in telecommunications [1], [7], [8]. Considering that 2D-PPs are evanescent (or dark) modes, they can neither radiate nor be excited directly by radiation. Therefore the capability of converting 2D-PPs into light and vice versa is particularly valuable for the aforementioned applications. For this reason alone, increasing the conversion efficiency is a pivotal challenge within the plasmonics and photonics communities [5], [9], [10], [11], [12], [13], [14], [15]. Moreover, with the advent of vdW heterostructures, extensive manipulation of the plasmonic properties became possible, both through stacking different types of 2D crystals that form a heterostructure, as well as through electrostatic or chemical doping [4], [16], [17]. Namely, the use of different 2D semiconducting layers enables the creation of diverse dielectric environments that could significantly change the plasmonic properties arising from conductive layers [18]. While previous theoretical work has focused on the intensity, dispersion [19], [20], [21], propagation length [22], [23] and damping of 2D plasmons [24], studies on the excitation efficiency of 2D-PP using external radiation are less common [10], [25], [26], [27]. Our work aims to deepen the understanding of light-2D-PP switching efficiency, particularly in vdW composites, which could enable on-demand selective coupling between light and 2D-PP, as mentioned above.

In this paper, we study the intensities of the electromagnetic modes in the vdW heterostructure composed of alternatively stacked doped graphene [Gr(n)] and hexagonal boron-nitride (hBN) single-layers (SL). Here *n* denotes the concentration of electrons in the graphene π* band. The electromagnetic modes are driven by a nearby silver (Ag) spherical nanoparticle (Ag-NP) illuminated by monochromatic radiation. The electronic response in hBN and Gr SLs are described using random phase approximation (RPA) optical conductivities *σ* ^0^ calculated from first principles [28], [29]. To describe the electromagnetic modes and their coupling to external radiation we applied the propagator technique; solving the Dyson equation for the propagator of the electromagnetic field (or photon propagator) Γ [29], [30], [31]. Here, the photon propagator Γ is the fundamental variable from which we derive all other studied quantities.

We briefly define the nomenclature of electromagnetic modes in a vdW composite composed of *N* graphene (Gr) and *N* − 1 hexagonal boron nitride (hBN) single layers (SLs), distinguishing one Dirac plasmon (DP) and *N* − 1 linear plasmons (LPs). We explore Ag-NP absorptivity when placed near the vdW composite, as well as the electrical fields scattered at screened Ag-NP and reflected from the vdW composite. We show that although Ag-NP is a very weak IR absorber, it channels the electromagnetic field very well into 2D-PP so that even 25 % of the incident field is converted into DP. We demonstrate the selective hybridisation of Ag-NP with DP of certain wavelength *λ*_*D*_, regardless of the doping *n* or the number of layers *N*. We also explore the spatial distribution of the electrical field carried by DP and LPs and explore the parameter space (*n*, *N*) for which the efficient excitation of LPs is feasible. Finally, we obtain a very good agreement with recent experimental measurements of the DP and LP in a Gr/hBN/Gr composite [32].

The paper is organized as follows: Section 2 outlines the system’s geometry and includes the derivation of the photon propagator Γ, RPA optical conductivity *σ* ^0^, and scattered electrical field *E* ^sc^. In Section 3 we present the computational details, followed by a discussion of results in Section 4. Finally, Section 5 summarizes our conclusions.

## 2 Theoretical formulation

**Figure 1:**
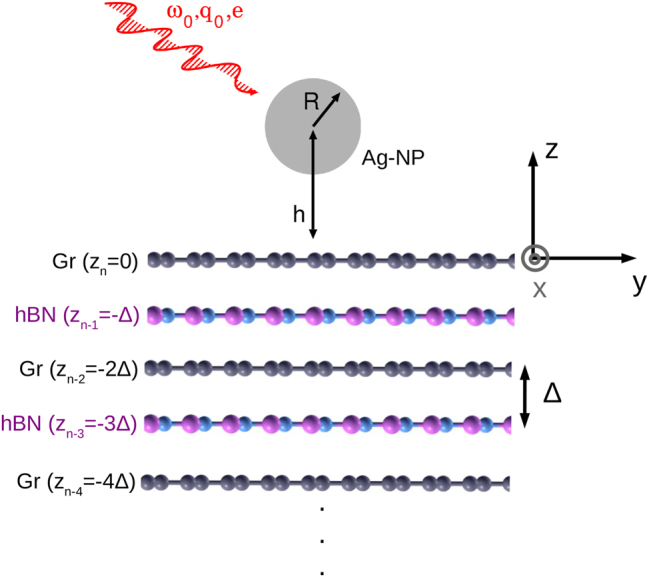
Geometry of the system. The electromagnetic modes in Gr/hBN composite are driven by a silver nanoparticle (Ag-NP) illuminated by monochromatic light of frequency *ω*_0_, wave vector **q**_0_ and of polarisation **e**.

In the scattering-type scanning near-field optical microscopy (s-SNOM) experiment the incident monochromatic radiation of frequency *ω*_0_ and wave vector **q**_0_ = (**Q**_0_, *q*_*z*0_), where **q**_0_ = *ω*_0_/*c*, excites localised dipole active modes (for example Mie resonances or plasmons) in the subwavelength AFM tip. This results in the scattering of the incident radiation to all radiative (far field) $\left|Q\right|<\omega_{0}/c$ and evanescent (near field) $\left|Q\right|>\omega_{0}/c$ partial waves which can excite corresponding electromagnetic modes in nearby vdW heterostructure. By imaging the scattered electrical field and its Fourier transform one obtains information about the electromagnetic modes in the vdW heterostructure. Here we study the electromagnetic modes in Gr/hBN heterostructure obtained by alternate stacking of *N* Gr and *N* − 1 hBN single-layers (SL), which occupy the planes *z*_*n*_ = 0, *z*_*n*−1_ = −Δ, *z*_*n*−1_ = −2Δ, …, as illustrated in Figure 1. For the separation between crystal planes, we have taken a constant value Δ. The AFM tip is here approximated by a silver spherical nanoparticle (Ag-NP) of radius *R* at height *h* relative to the topmost Gr layer, occupying *z* = 0 plane, as also illustrated in Figure 1. Before explaining the simulation of the s-SNOM experiment, i.e. the derivation of the scattered electric field or Ag-NP absorbance, we shall first derive the propagator the electric field Γ, as a starting variable from which all other values and conclusions are derived.

### 2.1 Calculation of photon propagator

Building upon the foundational research detailed in prior studies [28], [29], [30], [31], it is established that the intensity of electromagnetic modes in 2D crystals within the (**Q**, *ω*) phase space is determined by the real part of the electromagnetic field (or photon) propagator, denoted as $R\Gamma_{\mu \nu }\left(Q,\omega \right)$ . This procedure non-selectively scans all possible radiative $\left(\omega >\left|Q\right|c\right)$ and evanescent $\left(\omega <\left|Q\right|c\right)$ modes, yet it does not provide a measure of the actual excitation efficacy by external experimental probes. To address this gap, our research explores the efficiency with which external radiation can excite strong non-radiative modes, specifically plasmons, in conductive 2D crystals through the mediation of metallic spherical nanoparticles. In the following paragraphs, we show how this can be modelled by computing the scattered electrical field derived from the photon propagator Γ. We first assume the dielectric response the vdW composite is translationally invariant along the *x* − *y* plane, which means lateral crystal local field effects are neglected (**G**‖ = 0), while maintaining the dispersity of the dielectric response in the vertical *z* direction (*G*_*z*_ ≠ 0). In this context, **G** = (**G**‖, *G*_*z*_) represents 3D reciprocal vectors. Then the propagator Γ can be obtained by solving the following Dyson equation

(1) $$\begin{array}{ll} \Gamma_{\mu \nu }\left(Q,\omega ,z,z^{\prime }\right) & =\Gamma_{\mu \nu }^{0}\left(Q,\omega ,z,z^{\prime }\right)+\underset{\alpha \beta }{\sum }\overset{\infty }{\underset{-\infty }{\int }}dz_{1}dz_{2}\\ & \;\times \Gamma_{\mu \alpha }^{0}\left(Q,\omega ,z,z_{1}\right)\sigma_{\alpha \beta }^{0}\left(Q,\omega ,z_{1},z_{2}\right)\\ & \;\times \Gamma_{\beta \nu }\left(Q,\omega ,z_{2},z^{\prime }\right). \end{array}$$

Moreover, by applying a 2D approximation, the unscreened conductivity of a vdW composite, which consists of *n* parallel stacked 2D crystals that occupy the planes *z* = *z*_*i*_; ∀*i* ∈ [1, …, *n*], becomes

(2) $$\sigma_{\mu \nu }^{0}\left(Q,\omega ,z,z^{\prime }\right)=\overset{n}{\underset{i=1}{\sum }}\sigma_{\mu \nu }^{0,i}\left(Q,\omega \right)\delta \left(z-z_{i}\right)\delta \left(z^{\prime }-z_{i}\right),$$

where $\sigma_{\mu \nu }^{0,i}\left(Q,\omega \right)=\sigma_{\mu }^{0,i}\left(Q,\omega \right)\delta_{\mu \nu }$ represents the unscreened 2D conductivity in the *i*th 2D crystal. After we insert Eqs. (2) in (1) we obtain the following Dyson’s equation for Γ [31]

(3) $$\begin{array}{ll} \Gamma_{\mu \nu ij}\left(Q,\omega \right) & =\Gamma_{\mu \nu ij}^{0}\left(Q,\omega \right)+\underset{\alpha =x,y,z}{\sum }\overset{n}{\underset{k=1}{\sum }}\Gamma_{\mu \alpha ik}^{0}\left(Q,\omega \right)\\ & \;\times \sigma_{\alpha }^{0,k}\left(Q,\omega \right)\Gamma_{\alpha \nu kj}\left(Q,\omega \right), \end{array}$$

where ${\hat{\Gamma }}_{ij}^{0}\left(Q,\omega \right)={\hat{\Gamma }}^{0}\left(Q,\omega ,z_{i},z_{j}\right)$ and the bare electromagnetic field propagator is defined explicitly [28], [29], [33], [34] as

(4) $$\begin{array}{ll} {\hat{\Gamma }}^{0}\left(Q,\omega ,z,z^{\prime }\right) & =-\frac{4\pi i}{\omega }\delta \left(z-z^{\prime }\right)z\cdot z-\frac{2\pi }{\omega \beta }\\ & \;\times \left\{{\hat{E}}^{s}+{\hat{E}}^{p}\left(z,z^{\prime }\right)\right\}{\text{e}}^{\text{i}\beta |z-z^{\prime }|}. \end{array}$$

The s(TE) polarisation contribution is

(5) $${\hat{E}}^{s}={\left(\frac{\omega }{c\beta }\right)}^{2}\left[\begin{array}{ccc} \beta_{y}^{2} & -\beta_{x}\beta_{y} & 0\\ -\beta_{x}\beta_{y} & \beta_{x}^{2} & 0\\ 0 & 0 & 0 \end{array}\right],$$

while the p(TM) contribution is

(6) $${\hat{E}}^{p}\left(z,z^{\prime }\right)=\left[\begin{array}{ccc} \beta_{x}^{2} & \beta_{x}\beta_{y} & -Q\beta_{x}{\mathrm{sgn}}_{zz^{\prime }}\\ \beta_{x}\beta_{y} & \beta_{y}^{2} & -Q\beta_{y}{\mathrm{sgn}}_{zz^{\prime }}\\ -Q\beta_{x}{\mathrm{sgn}}_{zz^{\prime }} & -Q\beta_{y}{\mathrm{sgn}}_{zz^{\prime }} & Q^{2} \end{array}\right],$$

where sgn_*zz*′_ = sgn(*z* − *z*′), **Q** = *Q*(cos*θ*_***Q***_, sin*θ*_***Q***_), $Q=\sqrt{Q_{x}^{2}+Q_{y}^{2}}$ , *β*_*x*_ = *β* cos*θ*_***Q***_, *β*_*y*_ = *β* sin*θ*_***Q***_ and the complex wave vector in perpendicular (*z*) direction is $\beta =\sqrt{\frac{\omega^{2}}{c^{2}}-Q^{2}}$ . The RPA 2D conductivity of the *i*th 2D crystal can be split into intraband and interband contributions [28],

(7) $$\sigma_{\mu }^{0,i}\left(Q,\omega \right)=\sigma^{\text{intra}},i_{\mu }\left(\omega \right)\;+\;\sigma^{\text{inter}},i_{\mu }\left(Q,\omega \right).$$

Here the intraband (*n* = *m*) or Drude conductivity is,

(8) $$\sigma_{\mu }^{\text{intra}}\left(\omega \right)=i\frac{e^{2}}{m}\;\frac{n_{\mu }}{\omega +i\eta_{\text{intra}}},$$

where the effective number of charge carriers is

(9) $$n_{\mu }\;=\;-\frac{m}{Se^{2}}\;\underset{n}{\sum }\underset{K\in 1.\text{SBZ}}{\sum }\;\frac{\partial f_{nK}}{\partial E_{nK}}\;{\left|j_{nK,nK}^{\mu }\left(G=0\right)\right|}^{2}.$$

The interband (*n* ≠ *m*) conductivity is

(10) $$\begin{array}{ll} \sigma_{\mu }^{\text{inter}}\left(Q,\omega \right) & =-i\frac{\hbar }{S}\underset{n\neq m}{\sum }\underset{K\in 1.\text{SBZ}}{\sum }\;\frac{f_{nK}-f_{mK+Q}}{E_{nK}-E_{mK+Q}}\\ & \;\times \frac{{\left|j_{nK,mK+Q}^{\mu }\left(G=0\right)\right|}^{2}}{\hbar \omega +E_{nK}-E_{mK+Q}+i\eta_{\text{inter}}}, \end{array}$$

where the current vertices are defined as

(11) $$j_{nK,mK+Q}^{\mu }\left(G\right)=\underset{\Omega }{\int }dr{\text{e}}^{-\text{i}\left(Q+G\right)r}\;j_{nK,mK+Q}^{\mu }\left(r\right),$$

and the current produced by transitions between Bloch states $\left|nK\right\rangle \to \left|mK+Q\right\rangle$ is

$$\begin{array}{ll} j_{nK,mK+Q}^{\mu }\left(r\right) & =\frac{e\hbar }{2im}\left\{\phi_{nK}^{*}\left(r\right)\partial_{\mu }\phi_{mK+Q}\left(r\right)\right.\\ & \;\left.-\left[\partial_{\mu }\phi_{nK}^{*}\left(r\right)\right]\phi_{mK+Q}\left(r\right)\right\}. \end{array}$$

Here Ω = *S* × *l* is the normalization volume, *S* is the normalization surface, *l* is supercell lattice constant in the *z* direction (used in DFT calculation of the self-standing 2D crystal), (*n*, *m*) are band indices, **K** are 2D wave-vectors in the first surface Brillouin zone (1. SBZ) and $f_{nK}={\left[e^{\left(E_{nK}-E_{F}\right)/kT}+1\right]}^{-1}$ is the Fermi–Dirac distribution at a finite temperature *T*. It should be emphasized that all quantities in the equations below Eq. (7) should be superscripted with the index *i* because they refer to the *i*th self-standing 2D crystal, but we omit it for clarity.

The above 2D approximation makes it possible to express all studied quantities in terms of 2D conductivities $\sigma_{\mu }^{0,i}$ of self-standing 2D crystals which are stacked to form the vdW heterostructure. This procedure saves computational time and memory resources tremendously, but coincidentally still provides plausible results. However, the 2D approximation is completely justified only if the electronic band structures of neighbouring 2D crystals in the vdW composite weakly overlap, which also means that the layers of the same component should never be adjacent to each other [29], [30], [31]. For example, when Gr or transition metal dichalcogenides (TMDs) are stacked in multilayers the original band structure is not preserved. In the graphene bilayer, two extra parabolic bands appear at the K point, while the TMD bilayers usually transform into indirect band-gap semiconductors. As a result, the 2D approximation holds true for vdW heterostructures such as Gr/hBN/Gr/…, where the conductive zero-bandgap Gr layers are interspersed with wide-bandgap semiconductors such as hBN. In such configurations, the onset for the electron-hole excitations in the hBN spacer layers begins at *ω* ∼ *E*_*g*_, which does not interfere with Gr’s response at energies far below the gap *E*_*g*_. Considering that (due to hBN being a wide band gap semiconductor) the band structures of the individual layers (Gr and hBN) do not overlap at all near the bandgap, when Gr and hBN layers are stacked on top of each other, their band structure is replicated with minimal distortion – especially the shape of the *π* bands is conserved. This allows us to apply our 2D model without ambiguity.

We emphasize here that simpler analytical or semi-analytical TBA models could be used instead to obtain graphene and hBN optical conductivities [35], [36]. However, for lower doping and in the intermediate frequency range (0.5 < *ω* < 1.5 eV), this can reduce the accuracy of the results (as discussed in Section S5A of Supplementary Materials [37]). Therefore in this work the more accurate *ab initio* conductivities were calculated, as they are only slightly more computationally demanding for smaller unit cells, such as that of graphene or hBN.

### 2.2 Simulation of the s-SNOM experiment

Suppose that Ag-NP is illuminated by incident monochromatic radiation of unit amplitude

(12) $$E_{0}=e\;{\text{e}}^{\text{i}\left(q_{0}r-\omega_{0}t\right)}.$$

Since the wavelength of the incident radiation is much larger than the dimension of the nanoparticle (*λ*_0_ = 2*πc*/*ω*_0_ ≫ *R*) and also much larger than the unit cell of vdW composites (*λ*_0_ ≫ *a*, *l*), from now on we shall use the dipole approximation (**q**_0_ = 0). This is a standard procedure that does not affect the validity of the obtained results. The electromagnetic energy absorption rate in Ag-NP is then [38]

(13) $$A_{\text{NP}}\left(\omega_{0}\right)=\omega_{0}eI\alpha_{\mu \nu }\left(\omega_{0}\right)e,$$

where the Ag-NP screened (or dressed) polarizability tensor is

(14) $$\hat{\alpha }\left(\omega \right)=\left[\begin{array}{ccc} \alpha_{x}\left(\omega \right) & 0 & 0\\ 0 & \alpha_{x}\left(\omega \right) & 0\\ 0 & 0 & \alpha_{z}\left(\omega \right) \end{array}\right],$$

where

(15) $$\alpha_{\mu }\left(\omega \right)=\frac{\alpha_{0}\left(\omega \right)}{1+i\omega \alpha_{0}\left(\omega \right)\Gamma_{\mu \mu }^{\text{ind}}\left(\omega ,h\right)}.$$

Here the *bare* polarisability of Ag-NP is

(16) $$\alpha_{0}\left(\omega \right)=R^{3}\frac{\epsilon_{\text{Ag}}\left(\omega \right)-1}{\epsilon_{\text{Ag}}\left(\omega \right)+2},$$

where *ϵ*_Ag_(*ω*) represents the Ag macroscopic dielectric function, and *R* is the nanoparticle radius. The coupling strength between the plasmonic modes in Ag-NP and the electromagnetic modes in the vdW composite is

(17) $$\Gamma_{\mu \mu }^{\text{ind}}\left(\omega ,h\right)=\int \frac{\text{d}Q}{{\left(2\pi \right)}^{2}}\Gamma_{\mu \mu }^{\text{ind}}\left(Q,\omega \right){\text{e}}^{2\text{i}\beta h},$$

where *h* is the vertical distance of the nanoparticle from the origin *z* = 0, and the surface electromagnetic field propagator is defined as

(18) $$\Gamma_{\mu \nu }^{\text{ind}}\left(Q,\omega \right)=\Gamma_{\mu \nu nn}\left(Q,\omega \right)-\Gamma_{\mu \nu nn}^{0}\left(Q,\omega \right).$$

Here $\Gamma_{\mu \nu nn}^{0}$ and Γ_*μνnn*_ represent propagators of bare and screened electromagnetic fields in the topmost Gr layer (*z* = 0), respectively.

The reflected field propagator in Eq. (18) in principle represents the reflection coefficient of the entire vdW composite $r_{s,p}^{\text{vdW}}$ which can be expressed in terms of the reflectivities of individual layers (*i* = Gr, hBN) [34], [39]

$$\begin{array}{ll} r_{s}^{i}\left(Q,\omega \right) & =-\frac{2\pi \omega }{\beta c^{2}}\sigma_{x}^{i}\left(\omega \right)\\ r_{p}^{i}\left(Q,\omega \right) & =-\frac{2\pi }{\omega }\left[\beta \sigma_{y}^{i}\left(\omega \right)-\frac{Q^{2}}{\beta }\sigma_{z}^{i}\left(\omega \right)\right], \end{array}$$

where the screened conductivities are

$$\sigma_{\mu }^{i}\left(\omega \right)=\frac{\sigma_{\mu }^{0,i}\left(\omega \right)}{1-\Gamma_{\mu \mu }^{0}\sigma_{\mu }^{0,i}\left(\omega \right)};\;\mu =x,y,z,$$

and where the bare electrical field propagators are

$$\Gamma_{xx}^{0}=-\frac{2\pi \omega }{\beta c^{2}},\;\;\Gamma_{yy}^{0}=-\frac{2\pi \beta }{\omega },\;\;\Gamma_{zz}^{0}=-\frac{2\pi Q^{2}}{\beta \omega },$$

as described in detail in Section S5C of Supplementary Materials [37].

**Figure 2:**
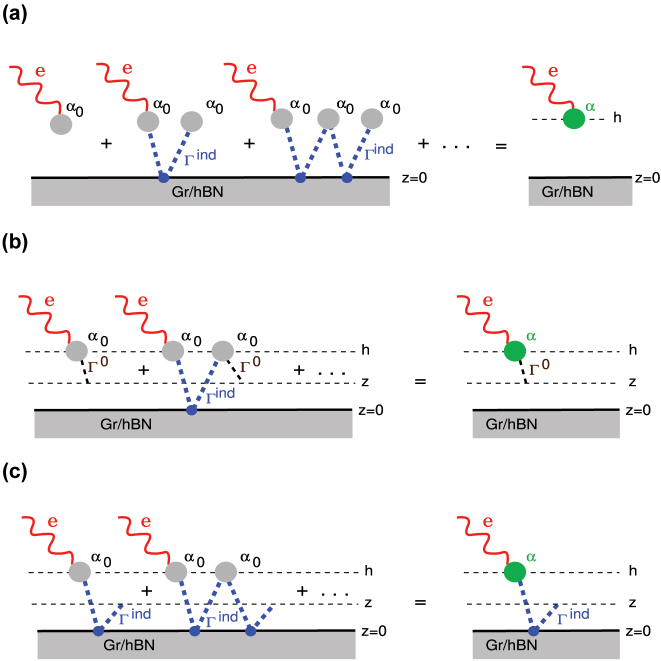
Schematic representation of (a) screened or dressed Ag-NP polarisability *α*; (b) the electrical field scattered on screened Ag-NP, **E** ^sc,vdW^ and; (c) the electrical field, first scattered at screened Ag-NP and then reflected from the vdW composite, **E** ^sc,vdW^. The latter field represents the 2D-PP fingerprint, induced by a screened dipole *α* **e**.

The derivation of the screened polarisability (Eq. (15)) is schematically illustrated in Figure 2(a). The Ag-NP dynamical dipole *α*_0_ **e** induces the electromagnetic field at the vdW surface which subsequently scatters and reflects multiple times generating a screened or dressed dynamical dipole $\hat{\alpha }e$ . The standard definition of electrical field at (***ρ***, *z*) driven by point dipole $p{\text{e}}^{-\text{i}\omega_{0}t}$ at (*ρ* = 0, *h*) is [29]

$$E\left(\rho ,z,\omega_{0}\right)=-i\omega_{0}\int \frac{\text{d}Q}{{\left(2\pi \right)}^{2}}{\text{e}}^{\text{i}Q\rho }\hat{\Gamma }\left(Q,\omega_{0},z,h\right)p,$$

where the propagator of the total electromagnetic field (propagating the interaction between *z* = *h* and 0 < *z* < *h*) is

(19) $$\hat{\Gamma }\left(Q,\omega_{0},z,h\right)={\hat{\Gamma }}^{0}\left(Q,\omega_{0},z,h\right)+{\text{e}}^{\text{i}\beta \left(z+h\right)}\Gamma_{\mu \nu }^{\text{ind}}\left(Q,\omega \right).$$

After inserting the definition of the bare propagator (Eqs. (4)–(6)), the total propagator (Eq. (19)) is explicitly

(20) $$\hat{\Gamma }\left(Q,\omega_{0},z,h\right)={\text{e}}^{\text{i}\beta h}\left[{\hat{\Gamma }}^{0}\left(Q,\omega_{0}\right){\text{e}}^{-\text{i}\beta z}+{\hat{\Gamma }}^{\text{ind}}\left(Q,\omega_{0}\right){\text{e}}^{\text{i}\beta z}\right],$$

where

(21) $${\hat{\Gamma }}^{0}\left(Q,\omega_{0}\right)=-\frac{2\pi }{\beta \omega_{0}}\left[\begin{array}{ccc} \frac{\omega^{2}}{c^{2}}-Q_{x}^{2} & -Q_{x}Q_{y} & Q\beta_{x}\\ -Q_{x}Q_{y} & \frac{\omega^{2}}{c^{2}}-Q_{y}^{2} & Q\beta_{y}\\ Q\beta_{x} & Q\beta_{y} & Q^{2} \end{array}\right].\;$$

Because in our case the dipole is the polarisable Ag-NP at (***ρ*** = 0, *h*) driven by external radiation, the following substitution can be performed

(22) $$p\;\to \;\hat{\alpha }\left(\omega_{0}\right)e,$$

and the scattered electrical field at (***ρ***, 0 < *z* < *h*) becomes

(23) $$E^{\text{sc}}\left(\rho ,z,\omega_{0}\right)=E^{\text{sc,NP}}\left(\rho ,z,\omega_{0}\right)+E^{\text{sc,vdW}}\left(\rho ,z,\omega_{0}\right).$$

The first term, schematically shown in Figure 2(b), represents the electromagnetic field scattered on the screened Ag-NP

(24) $$\begin{array}{ll} E^{\text{sc,NP}}\left(\rho ,z,\omega_{0}\right)= & -i\omega_{0}\int \frac{\text{d}Q}{{\left(2\pi \right)}^{2}}{\text{e}}^{\text{i}Q\rho }{\text{e}}^{\text{i}\beta \left(h-z\right)}\\ & \times {\hat{\Gamma }}^{0}\left(Q,\omega_{0}\right)\hat{\alpha }\left(\omega_{0}\right)e. \end{array}$$

The second term, depicted schematically in Figure 2(c), represents the field initially scattered by the screened Ag-NP and subsequently reflected from the vdW composite

(25) $$\begin{array}{ll} E^{\text{sc,vdW}}\left(\rho ,z,\omega_{0}\right) & =-i\omega_{0}\int \frac{\text{d}Q}{{\left(2\pi \right)}^{2}}{\text{e}}^{\text{i}Q\rho }{\text{e}}^{\text{i}\beta \left(h+z\right)}\\ & \;\times {\hat{\Gamma }}^{\text{ind}}\left(Q,\omega_{0}\right)\hat{\alpha }\left(\omega_{0}\right)e. \end{array}$$

More specifically, the first term represents the usual dipole electrical field which also takes into account the modification of the dipole polarisability due to the presence of the vdW surface. The second term represents the electrical field which produces the current in the vdW crystal induced by the screened dipole. In other words, the second term gives us information about the electric field carried by the 2D-PPs induced in the vdW crystal. Therefore, in the following, the 2D-PP will be analysed through the scattered field (Eq. (25)), and the modification of the Ag-NP polarizability will be analysed through the absorptivity (Eq. (13)). Moreover, it should be emphasized that the 2D-PP excitation efficiency strongly depends on screening affecting the polarisability *α*. We shall see below that it is the hybridization of Ag-NP with 2D-PP that enhances the polarisability in the infrared (IR) range and thereby increases the 2D-PP excitation efficiency. Lastly, we disregareded processes where the incident field initially scatters (or rather reflects) on the vdW composite. This omission is fully justified since the field reflected from the Gr/hBN composite is very weak, resulting in minimal scattering on the Ag-NPs and thus negligible excitation of 2D-PPs.

## 3 Computational details

The KS wave functions *ϕ*_*n* **K**_ and energies *E*_*n* **K**_ used to calculate the RPA conductivities *σ* ^0^ (Eqs. (7)–(11)) in Gr and hBN SLs were determined using a plane-wave self-consistent field DFT code (PWSCF) within the Quantum Espresso 6.4 package [40], [41], [42]. For both 2D crystals (Gr and hBN), as well as bulk Ag crystal, the core-electrons interaction was approximated by the norm-conserving pseudopotentials [43], [44] and the exchange-correlation (XC) potentials were approximated by the scalar-relativistic Perdew–Burke–Ernzerhof (PBE) generalized gradient approximation (GGA) functional [45]. The Gr and hBN ground state electronic densities were calculated by using the 12 × 12 × 1 Monkhorst–Pack (MP) K-mesh [46], the plane-wave cut-off energy was converged to 50 Ry and the density cut-off to 400 Ry. Both Bravais lattices were hexagonal, with cell parameters corresponding to *a*_Gr_ = 2.46 Å and *a*_hBN_ = 2.51 Å, respectively. The superlattice constant was *l* = 12.3 Å for both crystals. Optical conductivities of both graphene and hBN layers were calculated from Eqs. (7)–(11), where for hBN only the interband contribution (Eq. (10)) was taken into account. Therefore, the hBN conductivity was in fact calculated within the random phase approximation, while the ladder contribution *σ* ^ladder^, responsible for excitonic effects [29], was neglected. For both crystals the wave vector K summations in Eqs. (9) and (10) were performed by using a 201 × 201 × 1 K-mesh. The band summations were performed over 20 and 30 bands for Gr and hBN, respectively. Also, for both layers we used the same phenomenological damping constants *η*_intra_ = 10 meV and *η*_inter_ = 50 meV. The hBN band-gap was set to the DFT value $E_{\text{g}}^{\text{hBN}}=4.5\;\text{eV}$ . Here we assume that the vdW crystal and Ag-NP are surrounded by vacuum (i.e. *ϵ* = 1) and that Ag-NP is described by the macroscopic dielectric function

(26) $$\epsilon_{\text{Ag}}\left(\omega \right)=1/\epsilon_{G=0G^{\prime }=0}^{-1}\left(q\approx 0,\omega \right),$$

where **q** and **G** are the 3D transfer wave vector and the reciprocal lattice vector, respectively, corresponding to bulk Ag crystal. The dielectric matrix is $\hat{\epsilon }=\hat{\text{I}}-\hat{V}{\hat{\chi }}^{0}$ where ${\hat{\chi }}^{0}$ is 3D irreducible polarisability [30], [31] and bare Coulomb interaction is $V_{GG^{\prime }}\left(q\right)=\frac{4\pi }{|q+G{|}^{2}}\;\delta_{GG^{\prime }}$ . The ground state electronic densities of bulk Ag crystals were calculated by using the 8 × 8 × 8 K-mesh and the crystal structures were simulated by cubic FCC with lattice constants *a* = 4.143 Å. The converged plane-wave cut-off energy was 80 Ry. The wave vector **k** summation and band summations (*n*, *m*) in the *χ* ^0^ are performed using 81 × 81 × 81 *k*-meshes and over and 33 bands. The damping parameter was 40 meV  while the temperature was set to *T* = 10 meV and the crystal local field effects cut-off energy was set to be zero. For these parameters, the EELS spectrum $∼I1/\epsilon_{\text{Ag}}$ , exhibits a strong peak at *ω*_*p*_ ∼3.6 eV corresponding to Ag bulk plasmon [47]. Dielectric functions of noble metals (Ag, Au, Cu, …), due to the interband transitions between the multitude of *d* bands near the Fermi energy, deviate significantly from dielectric function of simple metals (Na, Li, Al, …) [48]. Therefore, the dielectric function of silver must be calculated using an *ab initio* model as demonstrated in Section S5B of Supplementary Materials [37]. Moreover, a very careful treatment of the *d* bands energy is required, otherwise the *d* interband continuum and thus the bulk and surface plasmons will have the wrong energies.

To ensure that the 2D approximation can be safely applied and therefore validate the results obtained, we performed ground state calculations of trilayer Gr/hBN/Gr composite. To ensure proper treatment of the long range London dispersion which is important to obtain the correct interlayer distance in the structural optimization, we employed the vdW-DF-cx functional [49]. The choice of pseudopotenials was the same as for hBN and Gr as noted above. The relaxation of atomic positions was performed self-consistently until forces on all atoms were below 1 mRy/a.u. while keeping the parallel (*a*, *b*) cell parameters strained to the graphene cell size as relaxing parallel cell parameters did not yield any difference in results. The perpendicular cell size was set to *c* = 7*a* to prevent spurious interaction occurring between periodic replicas. The distance between graphene and hBN layers was found to be Δ = 3.2 Å after the relaxation. The Brillouin zone was sampled with a 13 × 13 × 1 MP mesh and wavefunction/density cutoffs were converged to 65/280 Ry for the Gr/hBN/Gr trilayer. In addition, ground state calculations for Gr and hBN were also repated with the vdW-DF-cx functional for the band structure comparison with a 13 × 13 × 1 MP mesh. Their wavefunction/density cutoffs were converged to 55/240 Ry and 80/320 Ry, respectively. The Methfessel–Paxton electron smearing parameter was kept at 10 mRy for all calculations.

In the 2D model, the separation between Gr and hBN planes Δ was kept constant and set to 3.2 Å as obtained in the DFT structural optimization calculation for the Gr/hBN/Gr heterostructure. However, we emphasize that the results were quite insensitive to a small variation in Δ.

## 4 Results and discussions

To clarify the 2D plasmon mode nomenclature in the Gr/hBN composite we first analyse a very simple toy model. We start from the most simple model system – a single-layer doped graphene (*N* = 1). Moreover, we suppose that the electromagnetic modes propagate in the *x* direction **Q** = (*Q*, 0), taking into account that Gr is difficult to polarise in the *z* direction $\left(\sigma_{z}^{0}=0\right)$ , and neglecting polarisability due to interband excitations $\left(\sigma_{x,y}^{\text{inter}}=0\right)$ . In this case, Dyson’s equation (Eq. (3)) becomes

$$\hat{\epsilon }\hat{\Gamma }={\hat{\Gamma }}^{0}$$

where the dielectric tensor is the following 2 × 2 matrix

(27) $$\hat{\epsilon }=\left[\begin{array}{cc} \epsilon_{x} & 0\\ 0 & \epsilon_{y} \end{array}\right]$$

and where $\epsilon_{x}=1+\frac{2\pi \beta }{\omega }\sigma_{x}^{\text{intra}}$ and $\epsilon_{y}=1+\frac{2\pi \omega }{\beta c^{2}}\sigma_{y}^{\text{intra}}$ . Here, *ϵ*_*x*_ represents the dielectric response, and *ϵ*_*x*_ = 0 is the restoring force condition for the emergence of the p(TM) polarised mode (whose electrical field is parallel to its propagation, so that *ϵ*_*x*_ is also called the longitudinal dielectric function). *ϵ*_*y*_ represents the dielectric response, and *ϵ*_*y*_ = 0 restoring force condition for occurrence of s(TE) polarised mode (whose electrical field is perpendicular to its propagation, so that *ϵ*_*y*_ is also called the transverse dielectric function). In the evanescent region *ω* < *Qc*, after inserting Eq. (8) and taking the zero damping limit *η*_intra_ = 0, the longitudinal dielectric function becomes

(28) $$\epsilon_{x}=1-\tilde{\beta }\frac{\omega_{p}^{2}}{\omega^{2}},$$

whereas the transverse dielectric function becomes

(29) $$\epsilon_{y}=1+\frac{\omega_{p}^{2}}{\tilde{\beta }c^{2}},$$

where $\tilde{\beta }=\sqrt{Q^{2}-\frac{\omega^{2}}{c^{2}}}$ and $\omega_{p}=\sqrt{\frac{2\pi n_{x}e^{2}}{m}}$ . It’s important to mention that the quantity *ω*_*p*_ (because *n* is the surface charge density) does not have the dimension of frequency [1/time] but instead [ $\sqrt{\text{length}}$ /time]. However, we still keep this notation for consistency with past research (e.g. the bulk plasmon frequency is traditionally defined as $\omega_{p}=\sqrt{4\pi ne^{2}/m}$ ). Here we also leveraged the isotropic nature of graphene, where *n*_*x*_ = *n*_*y*_. Because (in this model) the condition *ϵ*_*y*_ = 0 never occurs, graphene does not support transverse eigenmodes. In the non-retarded limit, *c* → ∞ and $\tilde{\beta }\to Q$ , the condition *ϵ*_*x*_ = 0 is satisfied if

(30) $$\omega_{0}\left(Q\right)=\omega_{p}\sqrt{Q},$$

which is the dispersion relation of the longitudinal DP. A detailed analysis of Dirac plasmon intensity and scattered field in single-layer graphene (*N* = 1) for different doping concentrations *n* is presented in Section S2 of Supplementary Materials [37].

We now proceed to consider a more complex system, the Gr(n)/hBN/Gr(n) trilayer. Given that the Gr/hBN separation is defined by Δ, it follows that the Gr/Gr separation is 2Δ (see Figure 1). Because we assumed that both graphenes are equally doped and we neglected the interband polarisability (which means that the polarization of hBN is completely neglected), according to Dyson’s equation (Eq. (3)) the longitudinal dielectric tensor is

(31) $${\hat{\epsilon }}_{\text{L}}\left(Q,\omega \right)=I-\tilde{\beta }\frac{\omega_{p}^{2}}{\omega^{2}}\left[\begin{array}{cc} 1 & e^{-2\tilde{\beta }\Delta }\\ e^{-2\tilde{\beta }\Delta } & 1 \end{array}\right].$$

By solving the eigenvalue problem ${\hat{\epsilon }}_{\text{L}}E=0$ we obtain two plasmon modes, whose dispersion relations (in the non-retarded limit) are

(32) $$\omega_{\pm }\left(Q\right)=\omega_{0}\left(Q\right)\sqrt{1\pm e^{-2Q\Delta }},$$

and which produces the electrical field which in the neighbouring graphene sheets oscillates in-phase $E_{+}=\left(1,1\right)$ and out-of-phase $E_{-}=\left(1,-1\right)$ . Because in the long wavelength limit (*Q* → 0) one obtains $\omega_{+}\left(Q\right)\approx \omega_{p}\sqrt{2Q}$ and $\omega_{-}\left(Q\right)\approx \omega_{p}\sqrt{2\Delta }Q$ . Given that the 2D plasmon in graphene originates from intraband transitions within the band that forms a *cone* in the vicinity of the Dirac point (*K* point of the Brillouin zone), that band significantly shapes its dispersion so that this plasmon is identified as the Dirac plasmon (DP) [50]. In the case of multilayer graphene, the hybridization of 2D Dirac plasmons (in individual graphene layers) occurs, yielding a series of 2D plasmons whose count increases proportionally to the number of graphene layers. The plasmon with the largest (square-root-like) dispersion, in this case *ω*_+_, is a plasmon composed of coherent (in-phase) oscillations of Dirac plasmons in individual graphenes, and that is why this plasmon is also commonly referred to as the DP. Lower frequency plasmons have linear dispersion relations and are here referred to as linear plasmons and in this case *ω*_−_ is a LP. It should be emphasized that LPs arise as a result of the hybridization of DPs in individual graphenes and should not be confused with the definition of an acoustic plasmon (AP), the plasmon that arises as a result of the hybridization of a 2D plasmon and a surface plasmon [51] or as a result of the hybridization of two or more electronic plasmas of different Fermi velocities in the same or several bands within the same 2D crystal [52]. According to this toy model, in the long-wavelength limit *Q* ≪ 1/Δ, by increasing Δ (or hBN thickness) the LP phase velocity increases, while for DP it remains unchanged. However, for larger wave-numbers *Q* ≈ 1/Δ (see Eq. (32)) the DP still significantly depends on Δ. For example, for thicker layers the DP and AP degeneracy (*ω*_−_(*Q*) ≈ *ω*_+_(*Q*)) will occur for smaller wave vectors *Q* then for thinner layers (see Section S4 of Supplementary Materials [37]). In the s-SNOM experiment the excitation frequency is fixed at *ω*_0_ so that in SL Gr, according to Eq. (30), the light will scatter mostly into the wave vector

(33) $$Q_{p}=\omega_{0}^{2}/\omega_{p}^{2}.$$

Accordingly, in the Gr/Gr bilayer, the light will be scattered into two wave vectors *Q*_±_ which, according to Eq. (32), scaled to wave vector *Q*_*p*_, are

(34) $$\frac{Q_{-}}{Q_{p}}=\frac{1}{\sqrt{2\Delta Q_{p}}}∼\sqrt{\frac{n_{x}}{\Delta }}\;\text{and}\;\frac{Q_{+}}{Q_{p}}=\frac{1}{2}.$$

According to Eq. (34), the DP wave vector *Q*_+_ scaled to wave vector *Q*_*p*_ evidently displays universal behaviour so we predict that the peak at *Q*_+_/*Q*_*p*_ that appears in s-SNOM measurements is weakly dependent on Δ or graphene doping *n*. On the contrary, the peak at $\frac{Q_{-}}{Q_{p}}$ will increase with doping and decrease with Δ. To prove these claims we explore the Fourier transform of the scattered field at *z* = 0 driven by a *x* polarised unit point dipole at a height *h* relative to the Gr/hBN/Gr composite, which according to Eq. (25) and *α* **e** → **x** is

(35) $$E_{x}^{\text{sc,vdW}}\left(Q_{x},\omega_{0}\right)=-i\omega_{0}{\text{e}}^{\text{i}\beta h}\Gamma_{xx}^{\text{ind}}\left(Q_{x},\omega_{0}\right).$$

**Figure 3:**
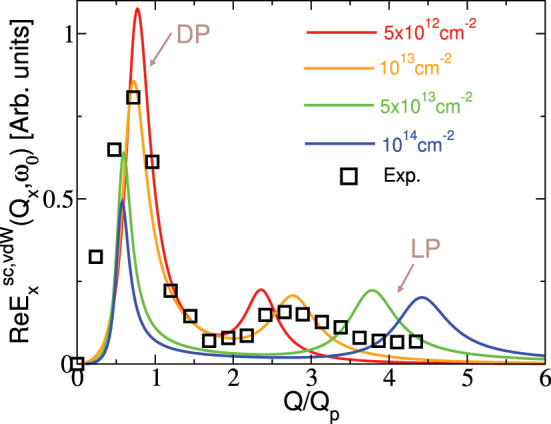
The Fourier transform of scattered electrical field $RE_{x}^{\text{sc,vdW}}\left(Q_{x},\omega_{0}\right)$ driven by a *x* polarised unit point dipole at a height *h* = 25 nm above the Gr(n)/hBN_37_/Gr(n) composite for different electron doping concentrations; *n* = 5 × 10^12^ cm^−2^ (red), *n* = 1 × 10^13^ cm^−2^ (orange), *n* = 5 × 10^13^ cm^−2^ (green) and *n* = 1 × 10^14^ cm^−2^ (blue). The experimental data was adapted from Ref. [32] and is represented by black squares.

In accordance with experiment [32] the excitation frequency is chosen to be *ℏω*_0_ = 117 meV, *h* = 25 nm and the Gr/hBN composite consists of 12 nm thick hBN dielectric slabs sandwiched by two graphene SLs. Adapted to our model the experimental system corresponds to Gr(n)/hBN_37_/Gr(n) composite consisting of 37 hBN layers and two Gr layers at a distance 2Δ ≈ 12.7 nm. Here we assumed that the hBN film is a bulk crystal so that hBN layers are 3.33 Å apart, the hBN-Gr distance at the bottom and top is 3.2 Å and both graphenes are equally doped by electrons of concentration *n*. Figure 3 shows the Fourier transform of the scattered field (Eq. (35)) in Gr(n)/hBN_37_/Gr(n) composite for different electron concentrations; *n* = 5 × 10^12^ cm^−2^ (red), *n* = 1 × 10^13^ cm^−2^ (orange), *n* = 5 × 10^13^ cm^−2^ (green) and *n* = 1 × 10^14^ cm^−2^ (blue). It can be seen that the change in the position of the DP peak is very small even though the doping is multiplied up to 20 times. On the contrary, the position of the LP peak moves significantly to the right as doping increases. The experimental result taken from Ref. [32] is represented by black squares. The experimental *Q*_*p*_ is also rescaled to correspond to our definition (Eq. (33)). Remarkably, we observer an excellent agreement with the experimental DP, even though the experimental doping is not known, confirming the universality of DP. Moreover, the consistency between our theoretical predictions and the experimental LPs allows us to estimate the experimental doping to be around *n* ≈ 1 × 10^13^ cm^−2^.

**Figure 4:**
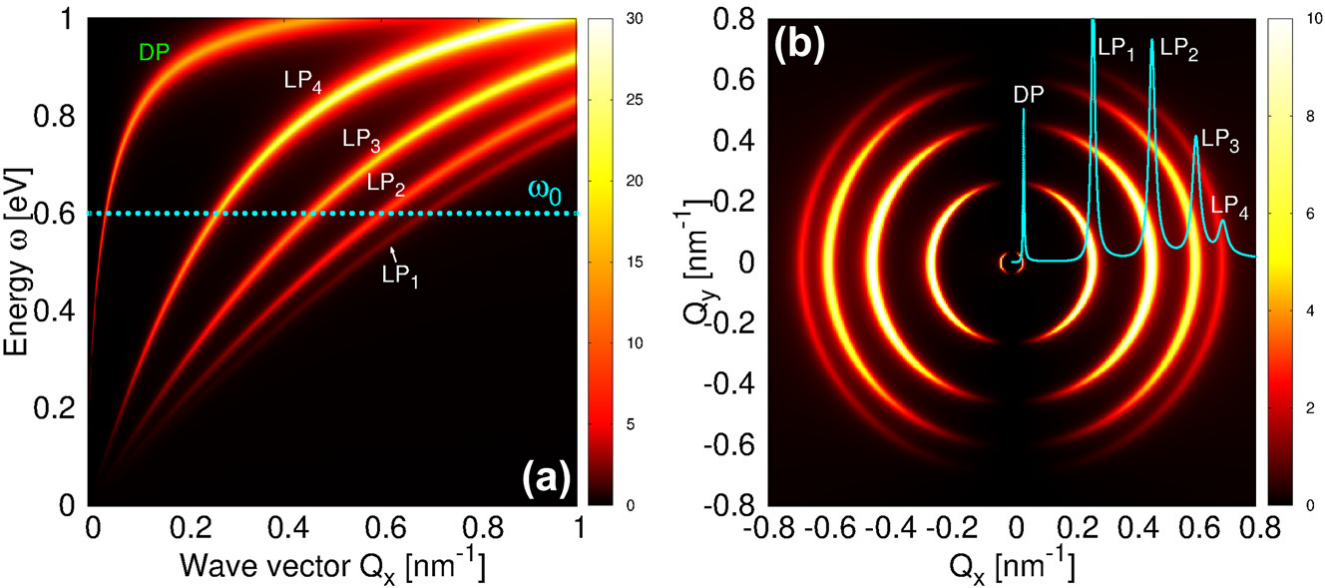
The intensity of the surface electromagnetic modes $-R\Gamma_{xx}^{\text{ind}}$ in Gr/hBN composite for *N* = 5 scanned over (a) (*Q*_*x*_, *ω*) space and over (b) (*Q*_*x*_, *Q*_*y*_) space for a fixed driving frequency *ω*_0_ = 0.6 eV denoted by a dotted horizontal line in (a). The solid cyan line in (b) represents the Fourier transform of scattered field, $E_{x}^{\text{sc,vdW}}\left(Q_{x},\omega_{0}\right)$ . Each Gr layer is doped by electrons of concentration *n* = 1 × 10^14^ cm^−2^. The intensities are in units $G_{0}^{-1}=h/2\pi e^{2}$ and the scattered field is in arbitrary units.

As the number of graphene layers increases, the number of linear plasmons multiplies, while the DP branch increasingly shifts towards higher energies. Figure 4(a) shows the intensity of the surface electromagnetic modes ( $-R\Gamma_{xx}^{\text{ind}}$ in units $G_{0}^{-1}=h/2\pi e^{2}$ ) in Gr(n)/hBN composite for *N* = 5 scanned over (*Q*_*x*_, *ω*) phase-space. Each Gr layer is doped by electrons of concentration *n* = 1 × 10^14^ cm^−2^. One can see a very steep peak corresponding to the DP and four linear plasmons LP_1_, LP_2_, LP_3_ and LP_4_ whose intensity can be even stronger than the DP. Figure 4(b) shows the intensity $-R\Gamma_{xx}^{\text{ind}}$ scanned over the (*Q*_*x*_, *Q*_*y*_) phase-space, for a fixed driving frequency *ω*_0_ = 0.6 eV, also denoted by a dotted horizontal line in Figure 4(a). We observe five concentric circular patterns which have the strongest intensity for $\left|\cos\theta_{Q}\right|\approx 1$ , while for $\left|\cos\theta_{Q}\right|\approx 0$ they vanish, indicating the longitudinal character of five plasmons. The solid cyan line in Figure 4(b) represents the Fourier transform of the scattered field $E_{x}^{\text{sc,vdW}}\left(Q_{x},\omega_{0}\right)$ (see Eq. (35)), which shows 5 peaks corresponding to 5 plasmons, exactly how they would appear in a SNOM measurement for this system. A detailed analysis of the dependence of the of the plasmon scattered field $E_{x}^{\text{sc,vdW}}$ w.r.t. doping *n* and the number of layers *N* is presented in Section S1 of Supplementary Materials [37].

In the subsequent paragraph, we will examine the efficiency with which external radiation, after scattering off Ag-NPs, couples to DP or LPs in various Gr/hBN composites.

**Figure 5:**
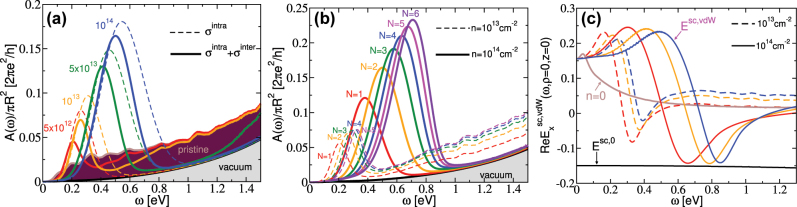
The normalised electromagnetic energy absorption rate in Ag-NP of radius *R* = 20 nm at height *h* = 30 nm from Gr(n)/hBN composite for (a) different doping concentrations; (brown) *n* = 0, (red) *n* = 5 × 10^12^ cm^−2^, (orange) *n* = 1 × 10^13^ cm^−2^, (green) *n* = 5 × 10^13^ cm^−2^; (blue) *n* = 1 × 10^14^ cm^−2^, for *N* = 2, and (b) for different thicknesses; (red) *N* = 1, (orange) *N* = 2, (green) *N* = 3, (blue) *N* = 4, (magenta) *N* = 5 and (violet) *N* = 6. The dashed and solid lines represent in (a) the Drude and full RPA results, respectively; and in (b) the results for electron concentrations *n* = 1 × 10^13^ cm^−2^ and *n* = 1 × 10^14^ cm^−2^ in the full RPA model, respectively. The absorption of Ag-NP in a vacuum (shaded in grey) is shown for comparison. (c) The dimensionless scattered field $E_{x}^{\text{sc,vdW}}$ at **r** = 0 in the Gr(n)/hBN composites of thicknesses (red) *N* = 1, (orange) *N* = 2 and (blue) *N* = 3 for Gr electron doping of *n* = 1 × 10^13^ cm^−2^ (dashed lines) and *n* = 1 × 10^14^ cm^−2^ (solid lines). The scattered field $E_{x}^{\text{sc,vdW}}\left(\omega \right)$ for *N* = 1 and *n* = 0 and field $E_{x}^{\text{sc,0}}$ (Eq. (24) where *α* → *α*_0_) are denoted by solid brown and black lines, respectively.

Figure 5(a) shows the normalised electromagnetic energy absorption rate in Ag-NP of radius *R* = 20 nm at a height *h* = 30 nm above the Gr(n)/hBN composite (*N *= 2) for different doping concentrations; *n* = 0 cm^−2^ (brown), *n* = 5 × 10^12^ cm^−2^ (red), *n* = 1 × 10^13^ cm^−2^ (orange), *n* = 5 × 10^13^ cm^−2^ (green) and *n* = 1 × 10^14^ cm^−2^ (blue). The dashed lines represent the results from the Drude model (*σ* ^0^ = *σ* ^intra^), while the solid line represents the results from the full RPA model (*σ* ^0^ = *σ* ^intra^ + *σ* ^inter^). The black solid line (shaded in grey) shows the absorption of Ag-NP in a vacuum. The absorbance of Ag-NP in a vacuum is, in the shown frequency range, negligible. The weak contribution we see is essentially the tail of the Mie plasmon resonance at *ω* ≈ 3.4 eV. However, when the Ag-NP is brought into close contact with the Gr/hBN composite, there is a marked increase in its absorbance, which exhibits a pronounced dependence on doping levels. In the case of pristine graphene (*n* = 0), there is a modest interaction between the Ag-NP and the interband *π* → π* excitations in Gr, resulting in relatively low absorbance – merely around 10 % of the quantum conductance of graphene $\left(\sigma_{0}=\frac{\pi e^{2}}{2h}\right)$ . However, for doped Gr layers, the absorbance exhibits broad maxima which increase both in intensity and frequency with doping. Specifically, the electronic plasma in the Ag-NP predominantly hybridizes with the Dirac plasmon (DP), and to a lesser extent with the linear plasmons (LPs) in the composite. This interaction results in the Ag-NP supporting not only the strong intrinsic Mie plasmon at 3.4 eV but also an additional, weaker infrared-active plasmon. In addition, as doping is increased, the DPs are becoming stronger and blue-shifted, so the infrared active plasmon follows the same trend. In the Drude model, where the DPs (without Landau damping) are much stronger, the absorption is also visibly stronger. Figure 5(b) shows the same as Figure 5(a) for different thicknesses; *N* = 1 (red), *N* = 2 (orange), *N* = 3 (green), *N* = 4 (blue), *N* = 5 (magenta) and *N* = 6 (violet). The dashed lines represent the results for electron concentration *n* = 1 × 10^13^ cm^−2^ and solid lines for *n* = 1 × 10^14^ cm^−2^. As the thickness of the composite increases, the spectral weight of the DP (coherent plasma oscillation along all Gr layers) increases, so that the hybridization with Ag-NP, and thus absorption, increases. A similar effect can be seen in Figure 5(a), because doping also raises the DP spectral weight. In Figure 5(a) and (b), we observe a notable universality: independent of the thickness *N* or doping level *n*, the Ag-NP consistently and primarily hybridizes with a specific group of Dirac plasmons (DPs). Specifically, the wavenumber of the DP, inferred from the peak absorption energy of the Ag-NP, is approximately $\left|Q\right|=Q_{\text{D}}∼0.052\;{\text{nm}}^{-1}$ , which corresponds to a DP wavelength of *λ*_*D*_ ∼ 120 nm. Lastly, the Ag-NP absorptivity, which reaches a maximum value of *σ*_0_, is still very weak.

The efficiency at which the external field is scattered on the Ag-NP or the vdW composite can be characterized by the ratio of incident and scattered fields *E* ^sc,NP,vdw^(*ω*)/*E*_0_. Given that the incident field Eq. (12) is assumed to have unit amplitude, it is sufficient to directly observe the fields *E* ^sc,NP,vdw^(*ω*), which are consequently also dimensionless. Figure 5(c) shows the dimensionless scattered electrical field $E_{x}^{\text{sc,vdW}}$ at **r** = 0 (which is exactly below the dipole in the *z* = 0 plane) in the Gr(n)/hBN composites of thickenesses *N* = 1 (red), *N* = 2 (orange) and *N* = 3 (blue), as well as Gr being doped at *n* = 1 × 10^13^ cm^−2^ (dashed lines) and *n* = 1 × 10^14^ cm^−2^ (solid lines). For reference, the scattered field $E_{x}^{\text{sc,vdW}}\left(\omega \right)$ for *N* = 1 and *n* = 0 and the field scattered on the unscreened Ag-NP $E_{x}^{\text{sc,0}}$ (Eq. (24) where *α* → *α*_0_) are denoted by solid brown and black lines, respectively. The polarization of the incident field is **e** = **x**.

The scattered field $E_{x}^{\text{sc,0}}\left(\omega \right)$ is structureless amounts to about 15 % of the incident field. The scattered field $E_{x}^{\text{sc, NP}}\left(\omega \right)$ was not shown because it differs negligibly from *E* ^sc,0^, i.e. screening does not affect the Ag-NP reactive response significantly. On the other hand, scattered fields $E_{x}^{\text{sc,vdW}}\left(\omega \right)$ (which can also be called reflected fields) exhibit both maxima and minima, right around the corresponding absorption maxima in Figure 5(a) and (b). This behavior is a definitive indicator of the excited DP field, a conclusion further supported by the absence of max/min behavior at a doping level of *n* = 0. Interestingly, in the static limit *ω* = 0, we observe the perfect screening regime, i.e. the scattered field $E_{x}^{\text{sc,0}}$ and the reflected field $E_{x}^{\text{sc,vdW}}$ cancel out. In other words, the dipole forms its own exact mirror image within the vdW heterostructure. Looking quantitatively, we see that (at the maximum) about 25 % of the incident field is converted into the DP, and this number does not vary significantly with either *N* or *n*. However, the conversion efficiency can be further modified by using different *h* and *R* parameters. Although the influence of nanoparticle size *R* on the plasmon excitation efficiency is very important, this dependence is almost trivial and scales with the volume of the nanoparticle, i.e. $E_{x}^{\text{sc,vdW}}\propto R^{3}$ . Namely, from Eq. (25) it can be seen that the excitation efficiency depends on *R* only through the Ag-NP screened polarisability *α*. But the vdW composite has a very weak effect on the Ag-NP bare polarisability, i.e. the approximation *α* ≈ *α*_0_ is fully justified. This can clearly be seen from the fact that the vdW composite has negligible influence Ag-NP absorptivity (see Figure 5(a) and (b)). Accordingly, this means that $E_{x}^{\text{sc,vdW}}$ is proportional to *α*_0_, and since *α*_0_ is, according to Eq. (16), proportional to *R* ^3^; $E_{x}^{\text{sc,vdW}}$ is apparently also proportional to *R* ^3^ (see Section S3A of Supplementary Materials [37]). Similar conclusions apply to the dependence of the scattered field $E_{x}^{\text{sc,vdW}}$ on the height parameter *h*. If we assume *α* ≈ *α*_0_, then *α* does not depend on *h*. Moreover, because the modes that contribute to the scattered field are mostly in the evanescent region (*ω* < *Qc*, *β* = *iβ*′ and $\beta^{\prime }=\sqrt{Q^{2}-\omega^{2}/c^{2}}\in R$ ), an exponential factor (e^−*β*^′^*h*^) appears under the integral in Eq. (25). Based on this, the scattered field should decrease exponentially (at least approximately) as the height parameter *h* is increased, for a fixed Ag-NP radius *R* (see Section S3B of Supplementary Materials [37]). In the following paragraphs, we will present the efficiency of DP and LP excitation and visualize them in real space.

**Figure 6:**
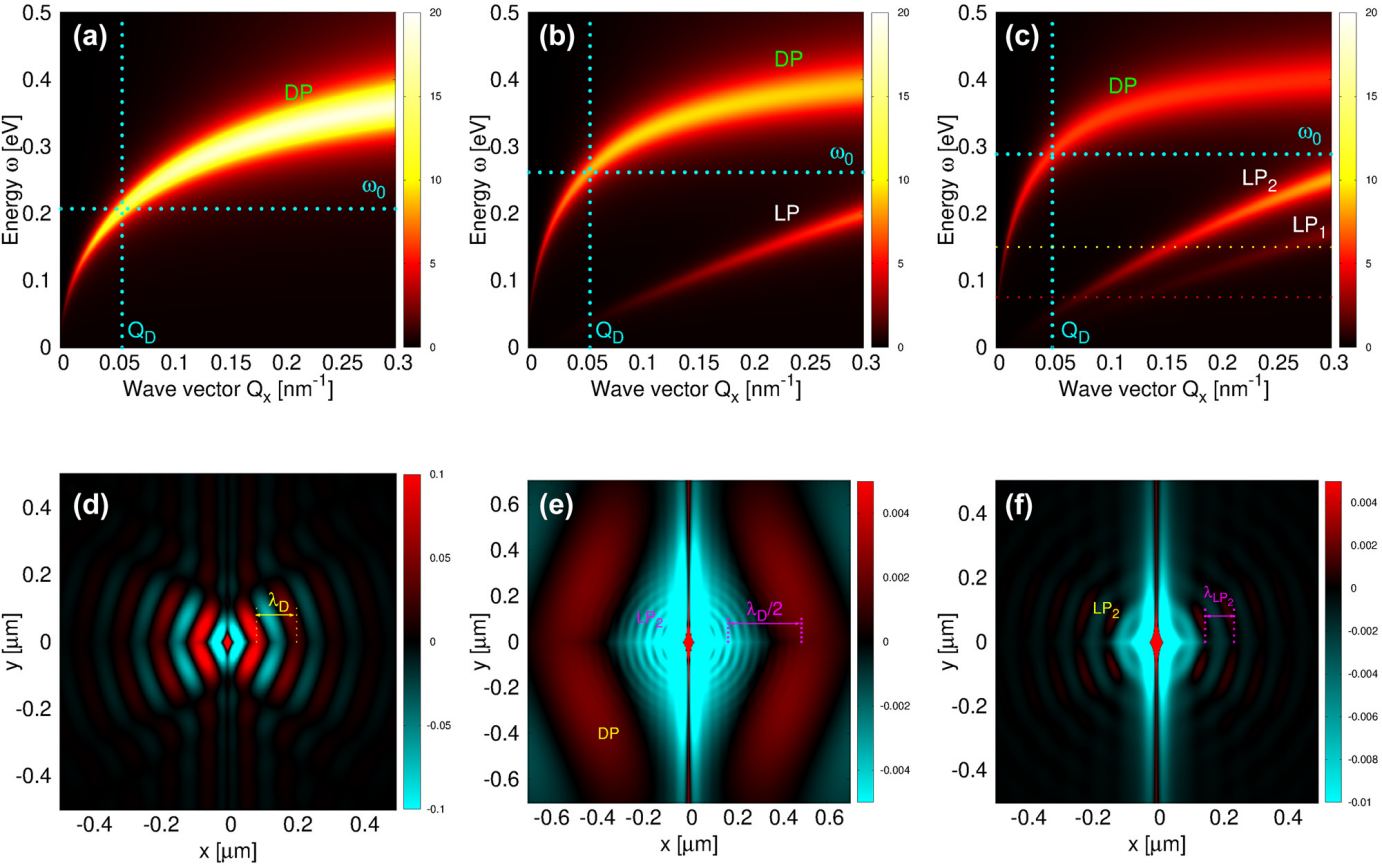
The intensities of the surface electromagnetic modes $-R\Gamma_{xx}^{\text{ind}}$ (in units $G_{0}^{-1}=h/2\pi e^{2}$ ) in gr(n)/hBN composite for (a) *N* = 1, (b) *N* = 2 and (c) *N* = 3. The dimensionless scattered field $E_{x}^{\text{sc,vdW}}\left(\rho ,z=0,\omega_{0}\right)$ driven by Ag-NP illuminated by *x* polarised radiation for (d) *N* = 1 and *ω*_0_ = 0.21 eV, (e) *N* = 3 and *ω*_0_ = 0.15 eV and (f) *N* = 3 and *ω*_0_ = 0.075 eV. Driving frequencies *ω*_0_ are also denoted by horizontal cyan line in (a) and yellow and red lines in (c). The graphene doping is *n* = 1 × 10^13^ cm^−2^.

**Figure 7:**
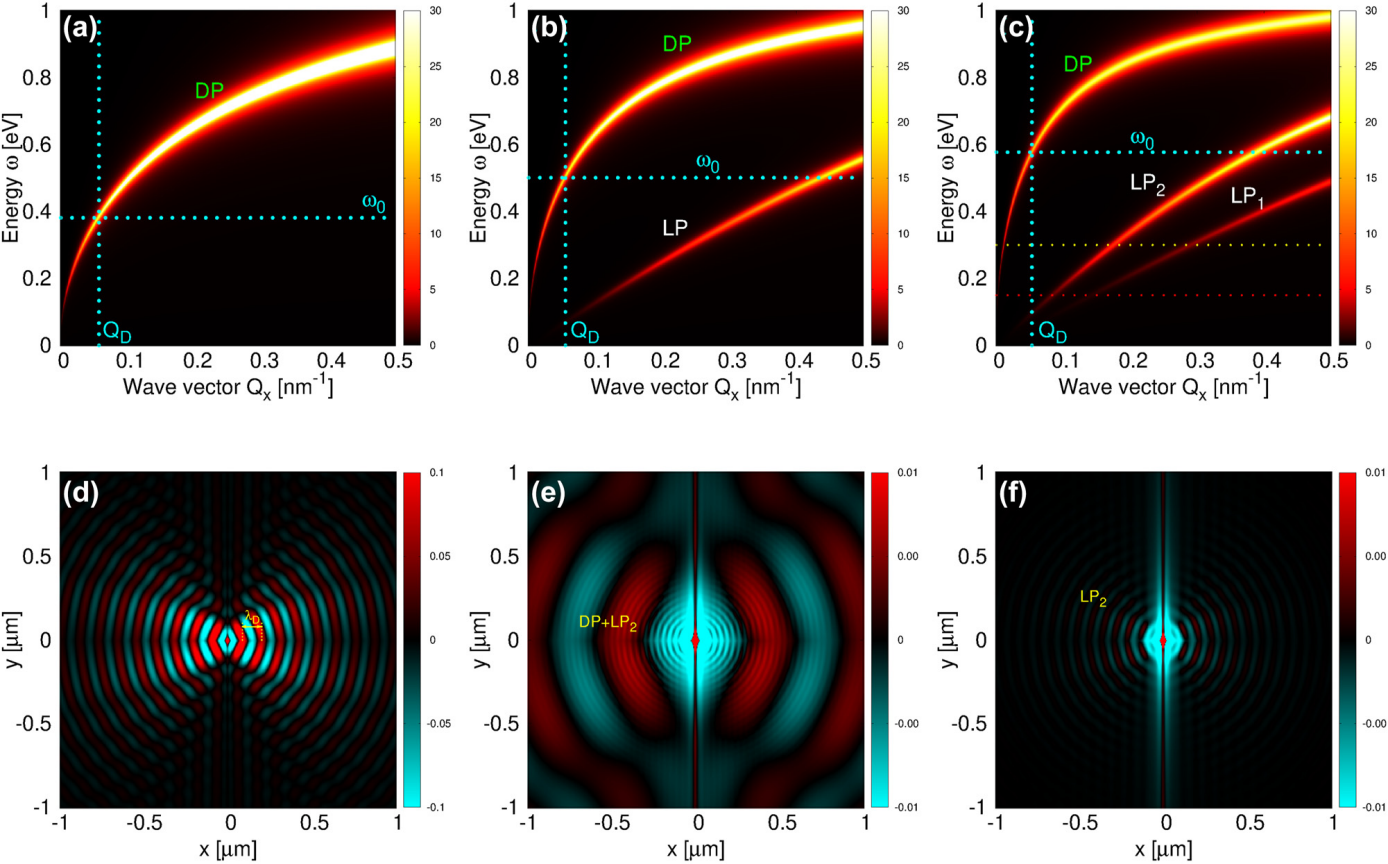
The intensities of the surface electromagnetic modes $-R\Gamma_{xx}^{\text{ind}}$ (in units $G_{0}^{-1}=h/2\pi e^{2}$ ) in gr(n)/hBN composite for (a) *N* = 1, (b) *N* = 2 and (c) *N* = 3. The dimensionless scattered field $E_{x}^{\text{sc,vdW}}\left(\rho ,z=0,\omega_{0}\right)$ driven by Ag-NP illuminated by *x* polarised radiation for (d) *N* = 1 and *ω*_0_ = 0.38 eV, (e) *N* = 3 and *ω*_0_ = 0.3 eV and (f) *N* = 3 and *ω*_0_ = 0.15 eV. Driving frequencies *ω*_0_ are also denoted by horizontal cyan line in (a) and yellow and red lines in (c). The graphene doping is *n* = 1 × 10^14^ cm^−2^.

The universality highlighted in Figure 5(a) and (b) is clearly illustrated in Figure 6(a)–(c), which displays the intensities of the surface electromagnetic modes $-R\Gamma_{xx}^{\text{ind}}$ for three different layer thicknesses (*N* = 1, 2, and 3) at a doping concentration of *n* = 1 × 10^13^ cm^−2^. This pattern is also evident in Figure 7(a)–(c) for a higher doping concentration of *n* = 1 × 10^14^ cm^−2^. In the six subfigures we denote by horizontal (cyan dotted) lines the excitation frequencies *ω*_0_ = 0.21 eV, 0.26 eV, 0.29 eV, 0.38 eV, 0.5 eV and 0.58 eV corresponding to Ag-NP absorption maxima in Figure 5(b). The DP wave vectors *Q*_*D*_ (also denoted by vertical dotted lines in Figures 6 and 7) corresponding to these frequencies are 0.055 nm^−1^, 0.055 nm^−1^, 0.050 nm^−1^, 0.057 nm^−1^, 0.055 nm^−1^ and 0.053 nm^−1^, respectively. Accordingly, it is obvious that the Ag-NP preferentially hybridizes with plasmons of wave number wave numbers $\left|Q\right|=Q_{D}∼0.052\;\text{nm−1}$ , corresponding to a wavelength *λ*_*D*_ ∼ 120 nm.

Figure 6(d) shows the dimensionless scattered field in the *z* = 0 plane $E_{x}^{\text{sc,vdW}}\left(\rho ,z=0,\omega_{0}\right)$ driven by Ag-NP illuminated by *x* polarised incident radiation of frequency *ω*_0_ = 0.21 eV (denoted by horizontal cyan line in Figure 6(a)), for *n* = 1 × 10^13^ cm^−2^ and *N* = 1. The parameters for the Ag-NP radius of *R* = 20 nm and a height of *h* = 30 nm were consistently applied throughout Figures 6(e) and (f) and 7(e) and (f). We observe a spreading wave pattern in the shape of a rhombus near the source, which later turns into circles. The wavelength, also denoted in this figure is exactly *λ*_*D*_ = 120 nm. The scattered fields corresponding to driving frequencies *ω*_0_ = 0.26 eV and 0.29 eV (denoted in Figure 6(b) and Figure(c)) are almost identical to the one shown in Figure 6(d), so we omitted those results for brevity. Figure 7(d) shows the same quantity as Figure 6(d), but for a driving frequency of *ω*_0_ = 0.38 eV (denoted in Figure 7(a)) and doping *n* = 1 × 10^14^ cm^−2^. We observe a propagating wave with a wavelength of *λ*_*D*_ = 116 nm, which, due to increased doping and consequently a greater oscillatory strength of the DP, extends well beyond the DP shown in Figure 6(d). Again, we omit the redundant results for the scattered field corresponding to the driving frequencies of *ω*_0_ = 0.5 eV and *ω*_0_ = 0.58 eV (denoted in Figure 7(b) and (c)). This concise analysis confirms that Ag-NP selectively excites plasmons of a specific wavelength, independent of the doping level *n* or the number of layers *N*.

For the above-selected excitation frequencies, the contribution of LPs in the scattered field is negligible as the Ag-NP excites LPs with large wave vectors *Q* ≫ 1/*h* inefficiently (due to the attenuation factor e^i*βh*^ ∼ e^−*Qh*^ in Eq. (25)). Therefore, to observe LPs, one should reduce the driving frequency *ω*_0_ enabling the excitations of LP with a smaller wave vector *Q*. Figure 6(e) and (f) show the scattered field $E_{x}^{\text{sc,vdW}}\left(\rho ,z=0,\omega_{0}\right)$ for *n* = 10^13^ cm^−2^, *N* = 3 and for driving frequencies *ω*_0_ = 150 meV and 75 meV, respectively. These driving frequencies are also denoted by yellow and red (horizontal, dotted) lines in Figure 6(c). In Figure 6(e) one can see circular waves around the source (forming broad wave minimum) and broad rhombic wave at around 450 nm from the source. These wave patterns represent LP_2_ (circular waves) modulated by the DP (rhombic wave). The wavelength of a circular wave $\lambda_{{\text{LP}}_{2}}∼40\;\text{nm}$ , and the wavelength of a rhombic wave *λ*_*D*_ ∼640 nm are consistent with Figure 6(c), where it can be seen that the yellow line intersects LP_2_ at *Q* ∼ 0.15 nm^−1^ (corresponding to $\lambda_{{\text{LP}}_{2}}∼42\;\text{nm}$ ) and the DP at *Q* ∼ 0.01 nm^−1^ (corresponding to *λ* ∼ 628 nm). Following previous conclusions, the circular wave patterns in Figure 6(f) undoubtedly represent LP_2_, while the DP is already very weak and its wavelength is far beyond the frame shown. The LP_2_ modulation can of course be amplified if we increase the Gr doping.

Figure 7(e) and (f) show the scattered field $E_{x}^{\text{sc,vdW}}\left(\rho ,z=0,\omega_{0}\right)$ for *n* = 1 × 10^14^ cm^−2^, *N* = 3 and for driving frequencies *ω*_0_ = 300 meV and 150 meV, respectively, also denoted by yellow and red lines in Figure 7(c). In Figure 7(e) one can clearly see LP_2_ (short wavelength patterns) modulated by the DP (long wavelength patterns). Figure 7(f) shows a weak LP_2_, which is, according to the red line in Figure 7(c), the only mode which can be efficiently excited at that frequency. The LP_1_ is not seen because, for selected frequencies, it is either very weak or has a large wave vector *Q* so that the dipole (whose field decreases as e^−*Qh*^) cannot excite it.

The way one can control the LPs excitation efficiency is by changing the thickness Δ. For example, for *N* = 2 the LP dispersion relation is $\omega_{-}\left(Q\right)\approx \omega_{p}\sqrt{2\Delta }Q$ (see Eq. (32)) so that by increasing Δ, the LP moves towards higher energies and smaller wave vectors *Q* and is therefore reachable by the dipole field. This was the case in Figure 3 when two Gr layers were separated by a thick hBN film. In case we want to efficiently excite more than one LP, we have to increase (as much as possible) the number of Gr layers *N* in the Gr/hBN composite. This could be the case already for *N* = 5. Considering the plasmon intensities shown in Figure 4, for the driving frequencies *ω*_0_ < 300 meV it is possible, following the conclusions relating to Figure 7(e), to excite even three linear plasmons; LP_2_, LP_3_ and LP_4_.

It is also important to consider that hBN supports polar LO phonon which can hybridize with Dirac plasmon in graphene sheets and thereby influence the electrodynamic properties of the vdW composite [53], [54]. In order to investigate how hybridization of LO phonons and plasmons affects the efficiency of Dirac plasmon launching, we performed a calculation in which hBN LO phonon is included via a local conductivity

$$\sigma_{\text{LO}}\left(\omega \right)=-\frac{i}{\pi }\frac{v_{\text{g}}\omega \omega_{\text{LO}}}{\omega_{\text{LO}}^{2}-\omega^{2}-i\omega \tau^{-1}}$$

described by three parameters: the LO phonon group velocity *v*_*g*_ = 1.2 × 10^−4^ c, frequency *ω*_LO_ = 1387 cm^−1^, and the phenomenological damping constant *τ* ^−1^ = 10 cm^−1^ [55]. Our calculations (see Section S6 of Supplementary Materials [37]) indicate that for dopings *n* ≥ 1 × 10^13^ cm^−2^ the oscillatory strength of the Dirac plasmon significantly prevails over the oscillatory strength of the LO phonon, so that the phonon weakly affect the plasmon spectrum and thus the plasmon excitation efficiency. Conceivably, for much lower doping values, when plasmon and photon oscillatory strengths become comparable, hBN phonon can more significantly affect the vdW composite electrodynamic properties. The same applies when graphene is physisorbed on a thicker hBN slab or at some insulating surface such as SiO_2_ [53], [54]. For example, in Ref. [56] it can clearly be seen that the Dirac plasmon (for *n* = 1 × 10^13^ cm^−2^) hybridizes far more weakly with the LO phonon in the hBN monolayer compared to the hybridisation with two SO phonons at the SiO_2_ surface.

In summary, the manipulation of composite thickness and graphene doping presents a versatile approach to modulate the excitation efficiency of 2D plasmons in vdW heterostructures. This tunability is pivotal for enhancing the performance of devices in photonics, plasmonics, and chemical sensing. While the silver nanoparticle (Ag-NP) utilized in our calculations primarily functioned as an efficient light scatterer rather than an absorber – owing to its plasmonic resonance in the UV region – it was instrumental in directing electromagnetic energy into both Dirac plasmons (DPs) and linear plasmons (LPs). Looking ahead, the substitution of Ag-NP with large organic molecules that support IR-active excitons – or leveraging IR-active molecular vibrational modes – could offer a more efficient method for directing radiation into various 2D plasmons. This approach may further optimize the plasmonic interactions for infrared applications, potentially leading to improved device performance in the fields of spectroscopy, optoelectronics, waveguiding, etc.

Finally, we note that retardation effects only have a minor impact on most conclusions of this work, especially for larger doping values (*n* ≥ 1 × 10^13^ cm^−2^) and the set of geometric parameters (*h*, Δ, *R*) we explored. Even though including retardation effects complicates the formalism somewhat and is computationally more demanding, we think it preferable (and justified) for two main reasons. Frist, even though for our selected geometric parameters the evanescent (near-field) effects are dominant, for other alternative geometries (e.g. larger *h* and/or Δ) radiative (far-field) effects would prevail rendering the non-retarded (*c* → ∞) formalism completely ineffective. Second, the retarded formalism treats p(TM) and s(TE) modes at the same level of accuracy, while in the non-retarded formalism s(TE) modes are not even accounted for. Therefore, although our study is limited to the study of p(TM) modes, extending it to s(TE) modes is straightforward, an option not available with the non-retarded approach.

## 5 Conclusions

In this study, we have demonstrated that subwavelength Ag nanoparticles (Ag-NPs) can efficiently funnel incident electromagnetic radiation into various 2D plasmons within a Gr/hBN heterostructure. We observed that with increased doping (*n*) and heterostructure thickness, up to 25 % of the incident electric field can be converted into a Dirac plasmon. Our results further reveal that external radiation can selectively excite a series of linear plasmons (LPs), with the degree of excitation controllable by adjusting the number of graphene layers (*N*) or the interlayer spacing (Δ). This tunability was corroborated by the strong correlation between our simulations of a graphene bilayer system (Gr/hBN_37_/Gr) and recent experimental data. A notable finding of our work was the observed universality in the interaction between Ag-NPs and DPs; Ag-NPs with specific dimensions (*R* and *h*) consistently hybridize with DPs of a particular wavelength (*λ*_*D*_), independent of *N* or *n*. This phenomenon enables targeted excitation of DPs by fine-tuning *R* and *h*. The ability to control these interactions through multiple parameters (*n*, Δ, *N*, *R* and *h*) holds significant potential for advancing plasmonics, photonics and optoelectronics, particularly in applications where selective and efficient photon-to-2D plasmon–polariton conversion is crucial.

## Supplementary Material

Supplementary Material Details
