# Supplementary material for: Optically driven plasmons in graphene/hBN van der Waals heterostructures: simulating s-SNOM measurements
Source: Nanophotonics. 2024 Apr 15;13(15):2765–80. doi: 10.1515/nanoph-2023-0841 (PMC11502017; doi:10.1515/nanoph-2023-0841)
Supplement: Supplementary file 1 — Supplementary Material Details [file j_nanoph-2023-0841_suppl_001.pdf]

Supplemental Materials for: Optically driven plasmons in Gr/hBN vdW heterostructures: simulation of s-SNOM measurements

Neven Golenić,[1,2] Stefano de Gironcoli,[2,3] and Vito Despoja[4,5,*]

[1]Department of Physics, University of Zagreb, Bijenika 32, 10000, Zagreb, Croatia
[2]Scuola Internazionale Superiore di Studi Avanzati (SISSA), Via Bonomea 265, 34136 Trieste, Italy
[3]CNR-IOM DEMOCRITOS, Istituto Officina dei Materiali, Trieste, Italy
[4]Centre for Advanced Laser Techniques, Institute of Physics, Bijenička 46, 10000 Zagreb, Croatia
[5]Donostia International Physics Center (DIPC),
P. Manuel de Lardizabal, 4, 20018 San Sebastin, Spain

S1. SCATTERED FIELD $E_x^{\mathrm{sc,vdW}}(Q_y,\omega)$ VERSUS DOPING CONCENTRATION n AND NUMBER OF GRAPHENE LAYERS N

Below we give a much more detailed description of the dependence of the plasmon scattered field on doping n and the number of layers N. Figure (S1) represents the Fourier transform of scattered electrical field driven by x polarised unit point dipole at height $h = 0$ from Gr(n)/hBN composite for four different electron concentrations; (a)$n = 5\times10^{12}\mathrm{cm}^{-2}$, (b)$n = 10^{13}\mathrm{cm}^{-2}$,(c)$n = 5\times10^{13}\mathrm{cm}^{-2}$ and (d)$n = 10^{14}\mathrm{cm}^{-2}$ and for different composites thicknesses (red) N=1, (orange) N=2, (green) N=3, (blue) N=4 and (violet) N=5. The driving frequency is ω_0=100meV. One can clearly see the peaks that represent the plasmons intensities, which multiplies as the number of layers increases. Also for higher doping $n > 5\times10^{13}\mathrm{cm}^{-2}$ plasmons are (as expected) shifted towards lower wave vectors Q_x and have significantly lower intensity. Figure (S2) represents the same as Fig.S1 but for the driving frequency $\omega_0 = 400$meV. For this driving frequency, the plasmons in the heavily doped composite have a much higher intensity, while in the the less doped samples ($n \le 10^{13}\mathrm{cm}^{-2}$), the plasmons are or already significantly suppressed by Landau damping or at lower frequency then excitation frequency ω_0, so that their excitation does not even occur [see e.g. Fig.(S2a)]. The Dirac plasmon is the most intense for the case of $N = 1$, however, its intensity decreases significantly as the number of layers increases. This is because its intensity centroid moves towards higher frequencies.

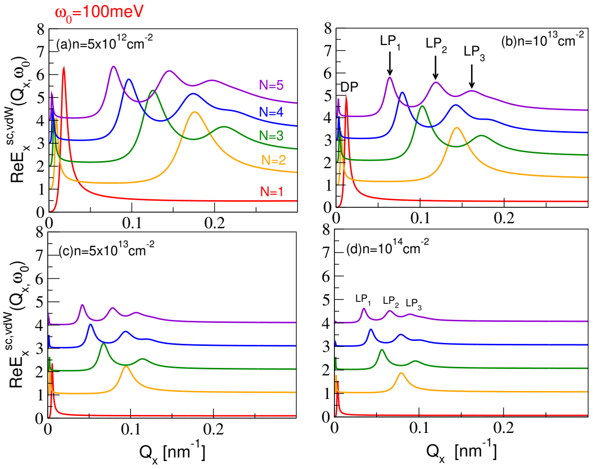

FIG. S1: Scattered field $E_x^{\mathrm{sc,vdW}}$ for four different electron concentrations; (a) $n = 5\times10^{12}\mathrm{cm}^{-2}$, (b) $n = 10^{13}\mathrm{cm}^{-2}$, (c)$n = 5\times10^{13}\mathrm{cm}^{-2}$ and (d) $n = 10^{14}\mathrm{cm}^{-2}$ and for different composites thicknesses (red) N=1, (orange) N=2, (green) N=3, (blue) N=4 and (violet) N=5. The driving frequency is ω_0=100meV

S2. DIRAC PLASMON EXCITATION EFFICIENCY IN SINGLE-LAYER DOPED GRAPHENE

Figures S3 show the intensity patterns (-Re$\Gamma_{xx}^{\mathrm{ind}}$) of Dirac plasmon and thus their frequencies (or dispersion relations) in single-layer graphene ($N = 1$) for different carrier densities (a)$n = 5\times10^{12}\mathrm{cm}^{-2}$, (b)$n = 10^{13}\mathrm{cm}^{-2}$,(c)$n = 5\times10^{13}\mathrm{cm}^{-2}$ and (d)$n = 10^{14}\mathrm{cm}^{-2}$. Figure S4 shows the scattered field in the graphene single layer for the same carrier densities n as in Figs.S3. The plasmons are driven by Ag-NP of radius $R = 20$nm at height $h = 30$nm from graphene. The insert show the frequency at which occur a maximum conversion of the incident field into a Dirac plasmon ($\omega_{\max}$) vs. carrier (doping) concentration n. One can notice that the $\omega_{\max}$ saturates approximately according to the square root law $\omega_{\max} \sim \sqrt{n}$. What is very interesting here is that the wave vector of the Dirac plasmon $Q_{\max}$ corresponding to $\omega_{\max}$ is always the same (or increases very slowly) regardless of the concentration n, which can be easily followed in the figures S3. In figure S4 we also show the scattered field for zero doping ($n = 0$). One can notice that even for zero doping scattered field shows small peak that apparently corresponds to the excitation of thermal Dirac plasmon.

*Electronic address: vdespoja@ifs.hr

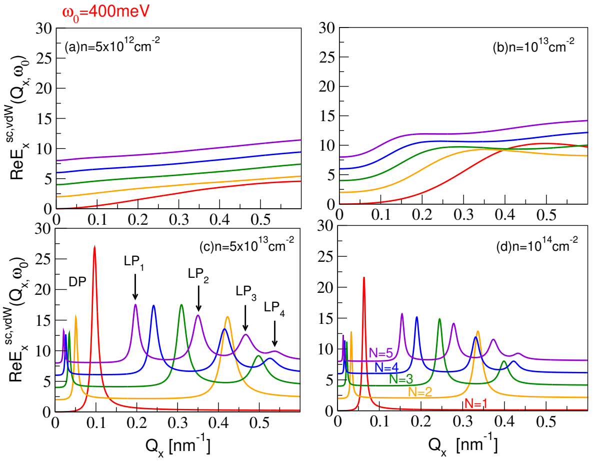

FIG. S2: The same as in Fig.S1 for driving frequency ω_0=400meV

S3. DIRAC PLASMON EXCITATION EFFICIENCY DEPENDENCE ON GEOMETRICAL PARAMETERS R AND h

A. Dirac plasmon excitation efficiency versus radius R

Although the influence of nanoparticle size R on the plasmon excitation efficiency is very important information here, this dependence is basically trivial and scales with the volume of the nanoparticle, i.e. $\mathbf{E}^{\rm sc,vdW} \propto R^3$. We will prove this below. Namely, the scattered field (or excitation efficiency) given by

$$\mathbf{E}^{\rm sc,vdW}(\boldsymbol{\rho}, z, \omega_0) = \\ -i\omega_0 \int \frac{d\mathbf{Q}}{(2\pi)^2} e^{i\mathbf{Q}\boldsymbol{\rho}} e^{i\beta(h+z)} \hat{\Gamma}^{\rm ind}(\mathbf{Q}, \omega_0) \hat{\alpha}(\omega_0)\mathbf{e}. \quad \text{(S1)}$$

depends on R only through the screened Ag-NP polarisability α. But the vdW composite has a very weak effect on the Ag-NP bare polarisability, i.e. it is also valid $\alpha \approx \alpha_0$. This is also clearly seen from the fact that the vdW composite negligibly influences the Ag-NP absorptivity [see Figs.5(a) and (b) in the main text]. This is especially true for the heights for which the AFM tip operates, i.e. $h > 20$nm. Accordingly, this means that $\mathbf{E}^{\rm sc,vdW}$ is proportional to α_0, and since α_0 is, according to equation (16) of the main text, proportional to R^3, $\mathbf{E}^{\rm sc,vdW}$ is apparently also proportional to R^3. Figure S5 shows the scattered field $E_x^{\rm sc,vdW}$ in the Gr(n)/hBN composites of thickness $N = 3$ and doping concentration $n = 10^{13}{\rm cm}^{-2}$ driven by Ag-NPs of different radii $R = 5$, 10, 15, 20, 25 and 30nm and for the fixed height $h = 30$nm. The insert shows the dependence of the scattered field maximum on the radius R (dots) and the corresponding R^3 fit (red line).

B. Dirac plasmon excitation efficiency versus height h

We can apply very similar conclusions when study the dependence of the scattered field $E_x^{\rm sc,vdW}$ on the parameter h. If we again assume that for $h \geq 20$nm $\alpha \approx \alpha_0$, then α does not depend on h. Moreover, because the modes that contribute to the scattered field are mostly in the evanescent region ($\omega < Qc$, $\beta = i\beta'$ and $\beta' = \sqrt{Q^2 - (\omega/c)^2} \in \mathbb{R}$), an exponential factor $e^{-\beta' h}$ appears under the integral in Eq.(S1). Based on this, the excitation efficiency should decrease exponentially (at least approximately) depending on the parameter h, for a fixed parameter R. Figure S6 shows the scattered field $E_x^{\rm sc,vdW}$ in the Gr(n)/hBN composites of thickness $N = 3$ and doping concentration $n = 10^{13}{\rm cm}^{-2}$ driven by Ag-NP of radius $R = 20$nm, for the different heights $h = 20$, 30, 40, 50, 60 and 70nm. The insert shows the dependence of the scattered field maximum on the height h (dots) and the corresponding ae^{-bh} fit (red line). One can notice a relatively good agreement with the exponential law, and the deviations appear only for larger h.

S4. DIRAC AND LINEAR PLASMONS (DP AND LP) IN GR/HBN-SLAB/GR COMPOSITE

For N = 2 and in the long-wavelength limit $Q << 1/\Delta$, where Δ is the distance between graphenes, the LP dispersion relation is $\omega_-(Q) \sim \sqrt{\Delta}Q$ and DP dispersion relation is $\omega_+(Q) \sim \sqrt{2Q}$ [see Eq.(32) in the main text]. Therefore, by increasing Δ (or hBN thickness) the LP phase velocity increases, however DP does not change much. However, for larger wave-numbers $Q \approx 1/\Delta$ the DP still significantly depends on Δ. For example, for thicker layers the DP and AP degeneracy [$\omega_-(Q) \approx \omega_+(Q)$] will occur for smaller wave vectors Q then for thinner layers. Figure S7(a) shows the intensity of the surface electromagnetic modes $-Re\Gamma^{\rm ind}_{xx}$ in Gr/hBN-SL/gr composite, and figure S7(b) in Gr/hBN-slab/gr composite where hBN-slab thickness is $\Delta = 5.3$nm, corresponding to 17 hBN layers. The graphene doping is $n = 10^{13}{\rm cm}^{-2}$. The dotted lines in figure S7(b) denote the dispersion relations of LP and DP in Gr/hBN-SL/gr composite, for comparison. One can see that for $Q << 1/\Delta$ the LP phase velocity significantly increases and DP does not change much. However for $Q = 1/\Delta \sim 0.2{\rm nm}^{-1}$, while in figure S7(a) DP and LP are still well separated, in figure S7(b) they degenerate.

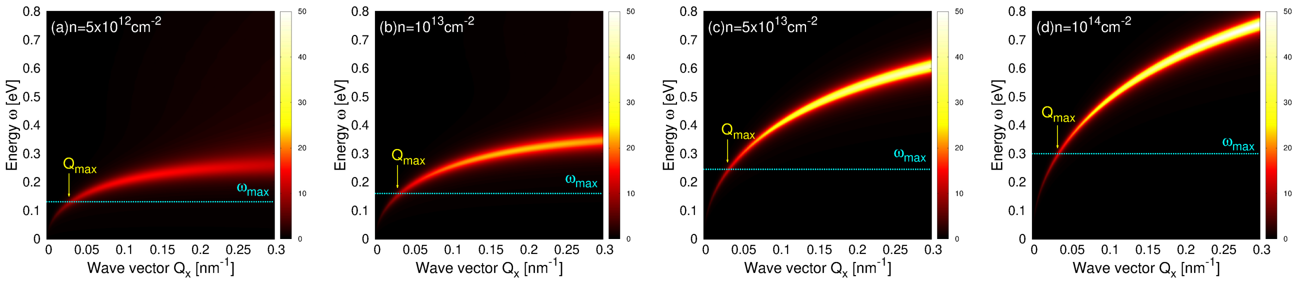

FIG. S3: The intensities of the electromagnetic modes (-ReΓ^{ind}_{xx}) in single-layer graphene ($N = 1$) for different carrier (doping) concentrations (a)$n = 5 \times 10^{12}\text{cm}^{-2}$, (b)$n = 10^{13}\text{cm}^{-2}$,(c)$n = 5 \times 10^{13}\text{cm}^{-2}$ and (d)$n = 10^{14}\text{cm}^{-2}$.

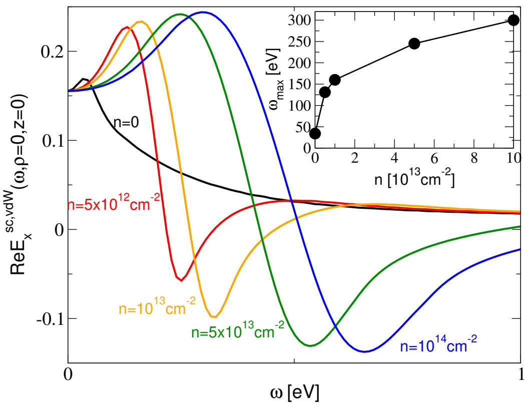

FIG. S4: The scattered field in the graphene single layer ($N = 1$) for the same carrier densities n as in Figs.S3. The insert show the frequency at which occur a maximum conversion of the incident field into a Dirac plasmon ($\omega_{\max}$) vs. carrier (doping) concentration n.

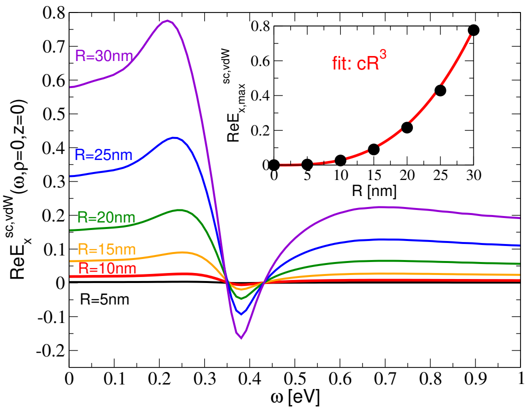

FIG. S5: The scattered field in the Gr(n)/hBN composites of thickness $N = 3$ and doping concentration $n = 10^{13}\text{cm}^{-2}$ driven by Ag-NPs of different radii $R = 5$, 10, 15, 20, 25 and 30nm and for the fixed height $h = 30$nm. The insert shows the dependence of the scattered field maximum on the radius R (dots) and the corresponding R^3 fit (red line).

S5. COMPARISON WITH THE RESULTS OF OTHER MODELS

A. *Ab initio* vs. TBA model

In the low frequency region we consider here ($\omega < 1.5$eV), for the graphene and hBN conductivities is probably sufficiently to use analytical expressions, such as the graphene conductivity in the Dirac cone approximation Eq.1 of Ref.[S1], rather than performing complex *ab initio* calculations. However, for lower doping and in the intermediate frequency range ($0.5 < \omega < 1.5$eV), this can reduce the accuracy of the results. In the following, we shall demonstrate this on the example of some of our result [Fig.5(c) in main text], where we compare the *ab initio* results and the results of the two-bands Tight Binding Approximation (TBA) model. Even, the TBA is still a slightly stronger approximation than the Dirac cone model[S1]. In the first-neighbor approximation, the best agreement between the *ab initio* and TBA model results was achieved for the hopping parameter $t = 2.1$eV and overlap matrix element $s = 0$[S2]. Figure S8(a) shows the conductivity of doped graphene for electron concentration $n = 10^{13}\text{cm}^{-2}$. Figure S8(b) shows the scattered field in Gr/hBN composite of thickness $N = 3$ for four doping concentrations (black)$n = 5 \times 10^{12}\text{cm}^{-2}$, (red)$n = 10^{13}\text{cm}^{-2}$, (blue)$n = 5 \times 10^{13}\text{cm}^{-2}$ and (violet)$n = 10^{14}\text{cm}^{-2}$. Dashed lines show TBA and solid lines show *ab initio* results. The TBA conductivity considerably underestimates the *ab initio* conductivity, but in the low-frequency range ($\omega < 1eV$) these deviations are not so large. However, these deviations are still large enough that the TBA results overestimate the *ab initio* results regarding the scattered electric field, as shown in figure S8(b). The analytical models would probably give an even larger deviation because it ignores the peak that appears in the conductivity at $\omega = 4$eV (which is a consequence of interband $\pi \to \pi^*$ transitions around the M point of Brillouin zone). So, although in this case the deviations are not very large, for some other choice of parameters it can be larger. Therefore, it is still safer to use more accurate *ab initio* conductivities, which are not demanding to calculate for smaller unit cells, such as

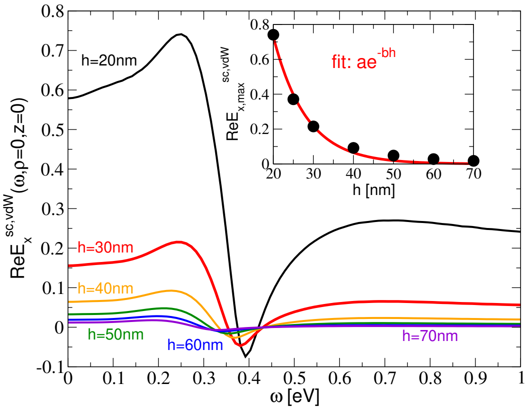

FIG. S6: The scattered field in the Gr(n)/hBN composites of thickness $N = 3$ and doping concentration $n = 10^{13}\text{cm}^{-2}$ driven by Ag-NP of radius $R = 20$nm, for the different heights $h = 20$, 30, 40, 50, 60 and 70nm. The insert shows the dependence of the scattered field maximum on the height h (dots) and the corresponding ae^{-bh} fit (red line)

graphene or hBN.

B. *Ab initio* vs. jellium (Drude) model

The dielectric functions of noble metals (Ag,Au,Cu,...) because of the interband transitions between the multitude of d bands near the Fermi energy deviates significantly from the dielectric function of simple metals (Al, Na, Li,...). In other words, the dielectric function of silver must be calculated using the *ab initio* model, and even more, a very careful treatment of the d bands energy centriod is required, otherwise the d interband continuum and thus the bulk and surface plasmons will have the wrong energies. Comparison of the *ab initio* (macroscopic) and Drude dielectric function for Wigner-Seitz radius $r_s = 3$ and damping parameter $1/\tau = 110$meV (adjusted to fit the *ab initio* intraband tail) in bulk silver is shown in figure S9(a). In Drude model the d interband continuum, that in *ab initio* model starts at about $\omega = 4$eV, is missing, which then greatly affects the position of surface plasmon and thus the Mie plasmon resonance in Ag-NP absorptivity, as can be seen in figure S9(b). The agreement between macroscopic *ab initio* dielectric function Fig.S9(a) and experimental dielectric function in bulk silver [S3] is also satisfactory good.

C. Propagator vs transfer matrix method

Our propagator formalism is fully equivalent to the transfer matrix method in which reflection or transmission coefficients are usually used[S4]. Below we compare two approaches to calculate the dipole radiation that scatters on the vdW heterostructure. One way is to simply calculate the propagator of the electric field Γ by solving the Dysons equation Eq.(1) of the main text. This way obtained $\Gamma(\mathbf{Q},\omega,z,z')$ represents the $(\mathbf{Q},\omega)$ component of the electric field at point z created by the point dipole at point z'. Another way is to use the local dielectric functions $\epsilon_i(\omega)$; $i =$gr,hBN and then to solve non homogeneous Maxwell's equations for the given boundary conditions. The first approach is much more elegant because: 1. The Dyson equation Eq.(1) enter the unscreened (or bare) conductivities $\sigma_\mu^{0,i}(\omega)$; $i =$gr,hBN; 2. these conductivities are already adapted to the 2D nature of the 2D crystals forming composite [see Eq.(2) of the main text] and 3. the obtained solution $\Gamma(\mathbf{Q},\omega,z,z')$ represents the radiation of a classical (rigid) dipole, so the extension to the radiation of a polarisable dipole (eg.the SNOM tip) is literally trivial [see Eq.(22) of the main text]. On the other hand, the transfer matrix method is much more appropriate when calculating the dipolar electric field in the vicinity of a composite of dielectric slabs of finite thicknesses [each described by a local dielectric function $\epsilon_i(\omega)$]. The transmitivities $t^i_{s,p}(\omega)$ and reflectivities $r^i_{s,p}(\omega)$ of each slab can be expressed using $\epsilon_i(\omega)$ and then, by means of recursive relations, the reflectivity of the entire composite is easily obtained from $t^i_{s,p}(\omega)$ and $r^i_{s,p}(\omega)$. In principle, the propagator method leads to the same result as the transfer matrix method. For example, each layer i can be described, instead by the bare conductivity $\sigma_\mu^{0,i}(\omega)$, by the reflectivity $r^i_{s,p}(\omega)$, which can be expressed in terms of the screened conductivity $\sigma^i_\mu(\omega)$ in the following way [S4, S5]:

$$r^i_s(Q,\omega) = -\frac{2\pi\omega}{\beta c^2}\sigma^i_x(\omega), \tag{S2}$$

$$r^i_p(Q,\omega) = -\frac{2\pi}{\omega}\left[\beta\sigma^i_y(\omega) - \frac{Q^2}{\beta}\sigma^i_z(\omega)\right]; \quad i = \text{gr,hBN}, \tag{S3}$$

where the screened conductivities are

$$\sigma^i_\mu(\omega) = \frac{\sigma^{0,i}_\mu(\omega)}{1 - \Gamma^0_{\mu\mu}\sigma^{0,i}_\mu(\omega)}; \; \mu = x,y,z, \tag{S4}$$

and where bare electrical field propagators are

$$\Gamma^0_{xx} = -\frac{2\pi\omega}{\beta c^2}, \;\; \Gamma^0_{yy} = -\frac{2\pi\beta}{\omega}, \;\; \Gamma^0_{zz} = -\frac{2\pi Q^2}{\beta\omega}. \tag{S5}$$

Furthermore, eg. for a two-component composite $i = 1,2$, the total reflectivity $r^{\text{double}}_{s,p}$ consists of four components, $r^{11}_{s,p}$, $r^{12}_{s,p}$, $r^{21}_{s,p}$ and $r^{22}_{s,p}$, where $r^{11}_{s,p} = r^1_{s,p}/(1 - r^1_{s,p}r^2_{s,p}e^{2i\beta\Delta})$, etc. If we now add a third component $r^3_{s,p}$, the new total reflectivity is constructed in a similar way, now using the reflectivities $r^{\text{double}}_{s,p}$ and $r^3_{s,p}$, etc. Basically, the total reflected field obtained in this way is identical to the reflected field Eq.(18) of the main text obtained by solving Dyson's equation Eq.(3). This can be easily proven for two-component system, eg. by using Eqs.(S2)-(S5) and the latter described procedure.

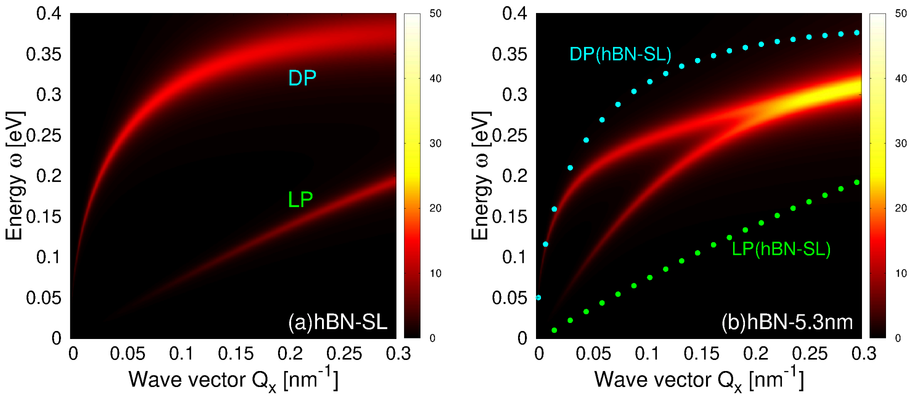

FIG. S7: The intensities of the surface electromagnetic modes $-Re\Gamma^{\rm ind}_{xx}$ in (a) Gr/hBN-SL/gr composite and in (b) Gr/hBN-slab/gr composite where hBN-slab thickness is $\Delta = 5.3$nm. The graphene doping is $n = 10^{13}{\rm cm}^{-2}$. The dotted lines in figure (b) denote the dispersion relations of LP and DP in figure (a), for comparison.

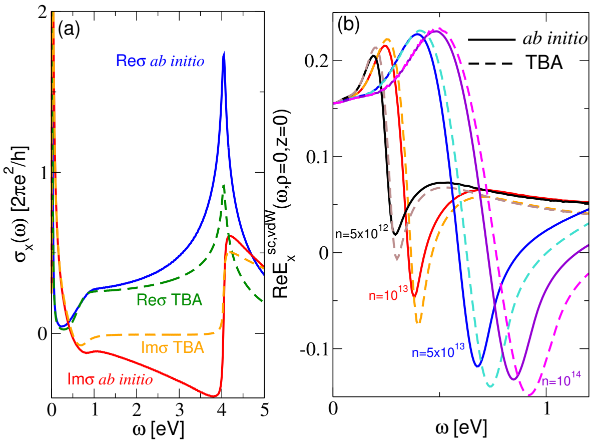

FIG. S8: (a) The conductivity of doped graphene for electron concentration $n = 10^{13}{\rm cm}^{-2}$. (b) The scattered field in Gr/hBN composite of thickness $N = 3$ for four doping concentrations (black)$n = 5\times10^{12}{\rm cm}^{-2}$, (red)$n = 10^{13}{\rm cm}^{-2}$, (blue)$n = 5\times10^{13}{\rm cm}^{-2}$ and (violet)$n = 10^{14}{\rm cm}^{-2}$. Dashed lines show TBA and solid lines show *ab initio* results.

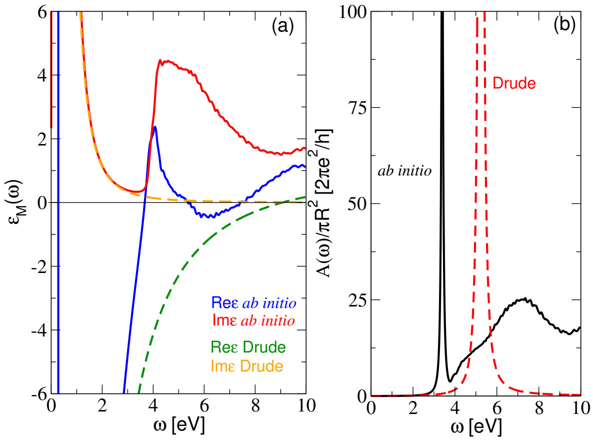

FIG. S9: (a)Comparison between *ab initio* (solid lines) and Drude (dashed lines) dielectric functions for Wigner-Seitz radius $r_s = 3$ and damping parameter $1/\tau = 110$meV (adjusted to fit the *ab initio* intraband tail) in bulk silver. (b)The normalised electromagnetic energy absorption rate in Ag-NP of radius $R = 20$nm obtained using *ab initio* (solid line) and Drude (dashed line) dielectric functions.

S6. EFFECT OF LO PHONON AND DP HYBRIDIZATION ON DP EXCITATION EFFICIENCY

In order to investigate how hybridization of LO phonons and plasmons affects the efficiency of Dirac plasmon launching, we performed a simulation in which hBN LO phonon is included via local conductivity

$$\sigma_{\rm LO}(\omega) = -\frac{i}{\pi}\frac{v_{\rm g}\omega\omega_{\rm LO}}{\omega^2_{\rm LO} - \omega^2 - i\omega\tau^{-1}}$$

described by three parameters; the LO phonon group velocity, frequency and the phenomenological damping constant $v_{\rm g} = 1.2\times10^{-4}c$, $\omega_{\rm LO} = 1387{\rm cm}^{-1}$ and $\tau^{-1} = 10{\rm cm}^{-1}$, respectively[S6]. Figure S10(a) shows the intensity of the electromagnetic modes (-Re$\Gamma^{\rm ind}_{xx}$) in Gr/hBN composite for doping concentration $n = 10^{13}{\rm cm}^{-2}$ and thickness $N = 3$ [corresponding to Fig.6(c) in the main text]. Figure S10(b) shows the zoom-in of the left lower corner in Fig.S10(a). Figure S10(c) shows the scattered field Re$E^{\rm sc,vdW}_x$ in Gr/hBN composite for $N = 3$ and for three doping concentrations $n = 5\times10^{12}$, and 10^{13} and $5\times10^{13}{\rm cm}^{-2}$. The inset zooms-in the region where the phonon effects appear. The solid lines includes the phonon effects and the dashed lines ignore it. What is immediately seen is that for dopings $n \geq 10^{13}{\rm cm}^{-2}$ the oscillatory strength of the Dirac plasmon significantly prevails the oscillatory strength of the LO phonon so that phonon weakly affects the plasmon spectrum and

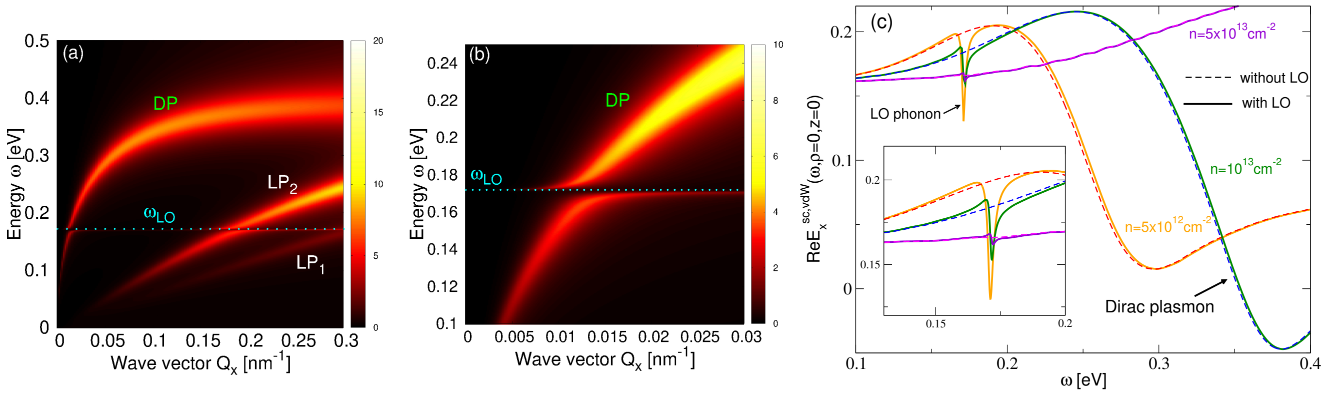

FIG. S10: (a) The intensity of the electromagnetic modes (-Re$\Gamma^{\rm ind}_{xx}$) in Gr/hBN composite for doping concentration $n = 10^{13}$cm^{-2} and thickness $N = 3$ and (b) shows the zoom-in of the left lower corner of a). (c) The scattered field Re$E_x^{\rm sc,vdW}$ in Gr/hBN composite for $N = 3$ and for three doping concentrations $n = 5 \times 10^{12}$, and 10^{13} and 5×10^{13}cm^{-2}. The inset zooms-in the region where the phonon effects appear. The solid lines includes the phonon effects and the dashed lines ignore it.

thus the plasmon excitation efficiency, as it can be seen in Fig.S10(c). Perhaps, for much lower dopings when plasmon and photon oscillatory strengths become comparable hBN phonon more significantly affect the vdW composite electrodynamic properties. The same applies when graphene is physisorbed at a thicker hBN slab, or at other insulating surfaces, such as SiO_2[S7, S8]. For example, in Ref.[S9] it can be clearly seen that the Dirac plasmon (for $n = 10^{13}$cm^{-2}) hybridizes much weaker with LO phonon in hBN monolayer than with two SO phonons at the SiO_2 surface.

[S1] Frank H. L. Koppens, D. E. Chang, and F. J. Garca de Abajo, Nano Lett. **11**, 3370 (2011)
[S2] I. Kupčić Phys. Rev. B **90**, 205426 (2014)
[S3] P. B. Johnson and R. W. Christy, Phys. Rev. B **6**, 4370 (1972)
[S4] M. S. Tomaš, Phys. Rev. A **51**, 2545 (1995)
[S5] Z. Rukelj, A. Štrkalj, and V. Despoja, Phys. Rev. B **94**, 115428 (2016)
[S6] N. Rivera, T. Christensen, and P. Narang, Nano Lett. **19**, 2653 (2019)
[S7] D. A. Iranzo, S. Nanot, Eduardo J. C. Dias, I. Epstein, C. Peng, D. K. Efetov, M. B. Lundeberg, R. Parret, J. Osmond, Jin-Yong Hong, J. Kong, D. R. Englund, N. M. R. Peres, F. H. L. Koppens, Science **360**, 291 (2018)
[S8] Eduardo J. C. Dias, D. A. Iranzo, P. A. D. Goncalves, Y. Hajati, Y. V. Bludov, Antti-Pekka Jauho, N. Asger Mortensen, F. H. L. Koppens, and N. M. R. Peres, Phys. Rev. B **97**, 245405 (2018)
[S9] V. W. Brar, M. S. Jang, M. Sherrott, S. Kim, Josue J. Lopez, L. B. Kim, M. Choi, and H. Atwater, Nano Lett. **14**, 3876 (2014)
